# A Review of Recent Advances towards the Development of (Quantitative) Structure-Activity Relationships for Metallic Nanomaterials

**DOI:** 10.3390/ma10091013

**Published:** 2017-08-31

**Authors:** Guangchao Chen, Martina G. Vijver, Yinlong Xiao, Willie J.G.M. Peijnenburg

**Affiliations:** 1 Institute of Environmental Sciences, Leiden University, 2300 RA Leiden, The Netherlands; vijver@cml.leidenuniv.nl (M.G.V.); xiao@cml.leidenuniv.nl (Y.X.); willie.peijnenburg@rivm.nl (W.J.G.M.P.); 2 Centre for Safety of Substances and Products, National Institute of Public Health and the Environment (RIVM), 3720 BA Bilthoven, The Netherlands

**Keywords:** nano-(Q)SARs, metallic nanomaterials, risk assessment, cellular uptake, toxicity

## Abstract

Gathering required information in a fast and inexpensive way is essential for assessing the risks of engineered nanomaterials (ENMs). The extension of conventional (quantitative) structure-activity relationships ((Q)SARs) approach to nanotoxicology, i.e., nano-(Q)SARs, is a possible solution. The preliminary attempts of correlating ENMs’ characteristics to the biological effects elicited by ENMs highlighted the potential applicability of (Q)SARs in the nanotoxicity field. This review discusses the current knowledge on the development of nano-(Q)SARs for metallic ENMs, on the aspects of data sources, reported nano-(Q)SARs, and mechanistic interpretation. An outlook is given on the further development of this frontier. As concluded, the used experimental data mainly concern the uptake of ENMs by different cell lines and the toxicity of ENMs to cells lines and *Escherichia coli*. The widely applied techniques of deriving models are linear and non-linear regressions, support vector machine, artificial neural network, *k*-nearest neighbors, etc. Concluded from the descriptors, surface properties of ENMs are seen as vital for the cellular uptake of ENMs; the capability of releasing ions and surface redox properties of ENMs are of importance for evaluating nanotoxicity. This review aims to present key advances in relevant nano-modeling studies and stimulate future research efforts in this quickly developing field of research.

## 1. Introduction

Manipulating matter at the nanoscale (1–100 nm) has provided a way forward to designing materials that exhibit inimitable magnetic, electrical, optical, and thermal properties compared to the bulk counterparts [1]. The products of engineered nanomaterials (ENMs) are consequently finding routine use in a wide range of commercial applications [2]. It was expected that the exponentially growing nano-market would reach a turnover of $65 billion by 2019 [3]. The release of ENMs into landfills, air, surface waters, and other environmental compartments therefore seems inevitable. In such a context, it is very likely for humans and for biota to encounter these nano-products and to be at risk given the potential adverse effects induced by ENMs. Studies on the cytotoxicity [4,5,6], neurotoxicity [7,8,9], genotoxicity [4,10,11], and ecotoxicity [12,13,14] of ENMs have shown that miniaturization of materials to the nanoscale may result in the appearance of evident ENM toxicity on organisms and human cell lines, which does not always occur at the bulk scales or cannot be well explained by the read-across of the properties of the bulk counterparts (i.e., the nano-specific effects). This highlighted the potential risks associated with the fast developing field of nanotechnology. Hence, seeking ways for the risk assessment of ENMs becomes imperative.

According to the commonly accepted procedures of chemical risk assessment, both exposure and hazard assessment are key to evaluating the risks of ENMs [15,16]. Hazard characterization, which aims at defining the dose-responses for targets or target-species is supposed to be mainly derived according to standardized test guidelines (e.g., Organization for Economic Co-operation and Development (OECD) guidelines). However, despite the existence of these powerful testing protocols, the possibility of covering all the existing and newly synthesized ENMs in the “nano pool” is reduced, taking into account the need of cost-effectiveness testing while minimizing the use of test animals. Considering the exponential increase of nanotechnology, the scarcity of data on ENM toxicity poses a major barrier to perform comprehensive hazard assessment of ENMs. As a result, the development of fast and inexpensive alternative approaches filling the data gaps and assisting in rationalizing ENMs’ risk assessment is of significant importance. Moreover, the principle of the 3R (replacement, reduction, and refinement) rule also calls for a reduction in the animal use and developing alternative non-animal testing approaches [17,18].

One of the most promising approaches that has long been particularly helpful for predicting biological effects of chemicals is the (quantitative) structure-activity relationship ((Q)SAR) method [19,20,21,22,23,24]. The (Q)SAR approach enables the encoding of existing knowledge into predictive models, which directly correlate the molecular structure with toxicity of a chemical. The role of (Q)SARs in predictive toxicology is [25,26] as follows:

- to provide fast and inexpensive high-throughput screening methods estimating the toxicity of chemical entity;
- to assist the classification of chemicals according to their toxicity;
- to help understand the underlying toxic mechanisms.

Two issues especially figure in the extraction of meaningful relationships between structures and biological effects to yield (Q)SAR models: the so-called molecular descriptor (measured or calculated) characterizing vital structural information of chemicals, and the so-called endpoint describing the biological effects of interest [27]. According to the OECD Principles for (Q)SAR Validation [28], it is essential for a (Q)SAR model considered suited for regulatory purposes to include information on (i) a defined endpoint; (ii) an unambiguous algorithm; (iii) a defined domain of applicability; (iv) appropriate measures of goodness-of-fit, robustness, and predictivity; and (v) a mechanistic interpretation, if possible.

Facing the strong need of extending the conventional (Q)SAR approach to nanotoxicology, some researchers have already made attempts to link ENMs’ biological effects with the characteristics of ENMs [29,30,31,32,33,34,35,36,37,38,39,40,41,42,43,44,45,46,47,48,49,50,51,52]. Given the large amount of reported laboratory-derived data on nanotoxicity and the many proposed nano-(Q)SARs, it is not very clear what data have been previously used by the modelers and what information on characterizing ENM structures was derived based on these data in previous studies. Doubt also exists as to what kinds of (Q)SAR-like models were previously introduced for ENMs. The employed descriptors in the nano-(Q)SARs are of special interest as they may contribute to a better interpretation of the mechanism of ENM biological profiles, such as the internalization of ENMs into cells and the interaction of ENMs with organelles. It is generally assumed that surface chemistry of ENMs is of significant importance for the uptake of ENMs into cells. The ions leached from ENMs and in some cases the nano-specific characteristics of ENMs play an important role in influencing nanotoxicity. Thus, based on these research questions, this paper reviews the state-of-the-art of the development of nano-(Q)SARs, for metal ENMs and metal-oxide ENMs, on the following aspects: (i) which data-sources are used for modeling; (ii) which different approaches are employed for deriving nano-(Q)SARs; (iii) based on the employed descriptors, what information can be obtained regarding the toxic mechanisms of the ENMs. At last, we present an outlook on the further avenues of development of nano-(Q)SARs.

## 2. Literature Search and Analysis

A literature search was performed by means of an Advanced Search in the Web of Science™ Core Collection on the 22 February 2017. The query for the literature search is ((((TS = (nano* AND metal)) AND (TS = (toxic*))) AND (TS = (quantitative *structure-activity relationship) OR TS = (*QSAR) OR TS = (QNAR) OR TS = (predict*) OR TS = (computation*) OR TS = (model*)))), where the field tag TS refers to the topic of a publication. From the search records, articles relevant to the development of nano-(Q)SARs predicting the biological activities of metal ENMs and metal oxide ENMs were extracted. The search of literature was manually supplemented with the relevant publications of interest not included in the search records. A summary of the retrieved literature is presented in Table 1 with brief description.

Based on these accessed information, the employed data for building models in the nano-(Q)SAR studies are firstly presented, including information on the original articles, the number of ENMs in the datasets, types of ENMs, and tested organisms in the experiments. These data are shown to be mainly from the assays of cellular uptake of metallic ENMs and the toxicity tests of metallic ENMs to various cell lines and *Escherichia coli* (*E. coli*). Information on characterizing the structures of ENMs based on experiment data in relevant studies are also described. Secondly, details on the workflows for model development and the resulting equations (if applicable) are subsequently summarized, with respect to the number of ENMs, descriptor calculation and selection, and the predictive performances of models. The widely employed statistical methods concluded from the state-of-the-art of nano-(Q)SARs are linear and logistic regressions, together with the approaches of support vector machines (SVM), artificial neural networks (ANN), *k*-nearest neighbors (*k*NN), etc. Additionally, the identified descriptors by the models reported are analyzed for interpreting the mechanisms of the biological activities of metallic ENMs.

## 3. Sources of Data for Modeling

As a data-driven approach, the field of nano-(Q)SARs highly relies on generating or assembling qualified experimental data. To integrate the existing information obtained from the various datasets that were successfully used in nano-QSARs, and therefore to aid further studies of nano-modeling, the underlying experimental data in the nano-(Q)SARs mentioned in Table 1 were analyzed. As can be seen in Table 2, research attention is found to be mainly on the cellular uptake of ENMs by different cell lines [53], on cytotoxicity [34,39,52,54,55], and on the toxicity of ENMs to *E. coli* [17,46]. Despite the numerous nano-related tests that are being carried out, it is to be concluded that only a few datasets (with data variety and consistency) were generally used as the data source for nano-(Q)SARs developed so far. The most widely applied data in (Q)SAR-like studies (Table 2) are from Weissleder et al. [53], Puzyn et al. [17], and Shaw et al. [54]. These experimental datasets are presented and arranged in the order of cellular uptake, cytotoxicity in cell lines, and toxicity to *E. coli* concerning the following aspects: (when available) types and numbers of ENMs, targets or target-species, toxicity endpoints, characteristics of the ENMs provided, and accessibility of relevant information.

### 3.1. Cellular Uptake Assays

Weissleder et al. [53] modified the surface of monocrystalline magnetic ENMs (3-nm core of (Fe_2_O_3_)_n_(Fe_3_O_4_)_m_) with 146 various small molecules (modifiers) and created a library of 146 water-soluble, magnetic, and fluorescent ENMs. ENMs were made magneto-fluorescent by adding the fluorescein isothiocyanate to the ENM surfaces. Uptake of these ENMs by five cell lines was screened afterwards. The cell lines used include pancreatic cancer cells (PaCa2), a macrophage cell line (U937), resting primary human macrophages, activated primary human macrophages, and human umbilical vein endothelial cells (HUVEC). A diversity of cellular uptake of various functionalized ENMs and a high dependence of ENM uptake on the composition of their surface were observed especially in the PaCa2 cells [30,56]. Data on PaCa2 cellular uptake of ENMs can be retrieved from Fourches’ studies [33,56] and the studies of Chau and Yap [30], Kar et al. [36], and Ghorbanzadeh et al. [35]. In the absence of data on calculated descriptors for the whole dataset, methods of characterizing ENMs in previous studies are presented in Table 3. An analysis of the methods reported in the literature shows that emphasis in ENM characterization is so far largely put on the characteristics of ENM surface modifiers, given the conclusion of Weissleder et al. [53] that the PaCa2 cellular uptake of ENMs highly depends on the surface modification of the ENMs. Descriptor calculation of the modifiers was performed within different software applications (e.g., PaDEL-Descriptor, DRAGON, ADRIANA) providing various molecular descriptors.

### 3.2. Toxicity to Various Cell Lines

One of the most widely used cell line-based toxicity data for ENMs is from the work of Shaw et al. [54]. In their study, four cell-based assays were performed based on four cell types at four different doses. The four types of cells, namely, endothelial cells (human aorta), vascular smooth muscle cells (human coronary artery), hepatocytes (human HepG2 cells), and murine RAW 264.7 leukemic monocyte/macrophage cells, were employed to assess the cytotoxicity of 50 ENMs (iron-based ENMs, pseudocaged ENMs, and quantum dots). The four cell-based assays were mitochondrial membrane potential, adenosine triphosphate (ATP) content, apoptosis, and reducing equivalents assays. Concentrations of 0.01, 0.03, 0.1, and 0.3 mg/mL Fe for iron-based ENMs, and 3, 10, 30, or 100 nM for quantum dots, were used. The ENMs were characterized by their coating, surface modification, size, the spin-lattice (R1) and spin-spin (R2) relaxivities, and the zeta potential. Experimental values were expressed in units of standard deviations of the distribution assessed when cells were only treated with PBS (*Z* score). Fourches et al. [33] afterwards transformed the 64 features (4 assays × 4 cell lines × 4 doses) of 48 iron-based ENMs into 1 by calculating their arithmetic mean (*Z*_mean_), which enabled binary classification studies based on this dataset (data are accessible in the original paper).

Gajewicz et al. [34] tested the cytotoxicity of 18 metal oxide ENMs to the human keratinocyte cell line (HaCaT). ENMs covered in the dataset include aluminum oxide (Al_2_O_3_), bismuth oxide (Bi_2_O_3_), cobalt oxide (CoO), chromic oxide (Cr_2_O_3_), ferric oxide (Fe_2_O_3_), indium oxide (In_2_O_3_), lanthanum oxide (La_2_O_3_), manganese oxide (Mn_2_O_3_), nickel oxide (NiO), antimony oxide (Sb_2_O_3_), silicon dioxide (SiO_2_), tin oxide (SnO_2_), titanium oxide (TiO_2_), vanadium oxide (V_2_O_3_), tungsten oxide (WO_3_), yttrium oxide (Y_2_O_3_), zinc oxide (ZnO), and zirconium oxide (ZrO_2_) ENMs. The cytotoxicity of these ENMs was characterized by cell viability of HaCaT and was expressed in terms of LC50 (concentration of the ENMs that leads to 50% fatality). Experimental data are accessible in the original publication. Moreover, 18 quantum-mechanical and 11 image descriptors were calculated for modeling purposes (Table 4). Information on the (aggregation) size for this dataset was provided by Sizochenko et al. [48] as shown in Table 5. Size (50 nm) and aggregation size (180 nm) of WO_3_ are not included in the table due to its absence in other datasets depicted in Table 5.

By measuring the plasma-membrane leakage via Propidium Iodide (PI) uptake in transformed bronchial epithelial cells (BEAS-2B), Liu et al. [39] studied the cytotoxicity of a variety of ENMs: Al_2_O_3_, cerium oxide (CeO_2_), Co_3_O_4_, TiO_2_, ZnO, copper oxide (CuO), SiO_2_, Fe_3_O_4_, and WO_3_ ENMs. The cytotoxicity was expressed in terms of percentage of membrane-damaged cells (data available in the supplemental information of the original publication). Descriptors calculated include the number of metal and oxygen atoms (*N*_Metal_ and *N*_Oxygen_), the atomic mass of the ENM metal (*m*_Me_), the molecular weight of the metal oxide (*m*_MeO_), the group and period of the ENM metal (*G*_Me_ and *P*_Me_), the atomization energy of the metal oxide (*E*_MeO_), the ENM primary size (*d*), the zeta potential, and the isoelectric point (IEP).

Another dataset that was provided by Zhang et al. [52] contains information on the toxicity of 24 oxide ENMs: Al_2_O_3_, CuO, CeO_2_, Co_3_O_4_, CoO, Cr_2_O_3_, Fe_2_O_3_, Fe_3_O_4_, gadolinium oxide (Gd_2_O_3_), hafnium oxide (HfO_2_), In_2_O_3_, La_2_O_3_, Mn_2_O_3_, NiO, Ni_2_O_3_, Sb_2_O_3_, SiO_2_, SnO_2_, R-TiO_2_, WO_3_, Y_2_O_3_, ytterbium oxide (Yb_2_O_3_), ZnO, and ZrO_2_ ENMs (data available in the original paper). The toxicity was expressed in terms of logEC_50_, in which EC_50_ means the effective concentration that causes 50% response. The lactate dehydrogenase (LDH), 3-(4,5-dimethylthiazol-2-yl)-5-(3-carboxymethoxyphenyl)-2-(4-sulfophenyl)-2H-tetrazolium (MTS), and ATP assays were implemented to assess the nanotoxicity to BEAS-2B and RAW264.7 cells in the study. Information on the crystalline structure of the ENMs (crystal system, space group, and unit cell parameters), primary and hydrodynamic sizes of metal oxide ENMs, and parameters for calculating ENM band energies (conduction and valence band, band gap energy, absolute electronegativities, and point of zero zeta-potential) were also provided by these authors. Liu et al. [41] built a nano-SAR model based on these data along with a summary of the calculated physicochemical properties of the ENMs. Information on 13 descriptors was provided including the ENM primary size (*d*), the energy of the conduction band (*E*_C_), the energy of the valence band (*E*_V_), the metal oxide atomization energy (*E*_Amz_), the metal oxide electronegativity (χ_MeO_), the metal oxide sublimation enthalpy (∆*H*_sub_), the metal oxide ionization energy (∆*H*_IE_), the metal oxide standard molar enthalpy of formation (∆*H*_sf_), the metal oxide lattice enthalpy (∆*H*_Lat_), the first molar ionization energy of metal (∆*H*_IE,1+_), the ionic index of metal cation (*Z*^2^/*r*), the IEP, and the zeta potential in water at a pH of 7.4 (ZP). Data of these descriptors can be accessed in the relevant articles.

### 3.3. Toxicity to E. coli

Puzyn et al. [17] tested the toxicity of 10 metal oxide ENMs to an *E. coli* (Migula) Castellani & Chalmers (ATCC#25254) strain. Metal oxide ENMs covered in the test are Bi_2_O_3_, CoO, Cr_2_O_3_, In_2_O_3_, NiO, Sb_2_O_3_, SiO_2_, V_2_O_3_, Y_2_O_3_, and ZrO_2_ ENMs. Meanwhile, results of another 7 metal oxide ENMs tested with the same protocol, namely, Al_2_O_3_, CuO, Fe_2_O_3_, La_2_O_3_, SnO_2_, TiO_2_, and ZnO ENMs, were taken from the study by Hu et al. [57], and a dataset consisting of 17 metal oxide ENMs was built. Toxicity to *E. coli* was expressed in terms of the logarithmic values of molar 1/EC_50_ in the original article. Data are shown in Table 5.

Meanwhile, information on the characterization of these ENMs in the reported nano-QSARs is presented in light of integrating existing resources and offering reference. As shown in Table 5, Kar et al. [37] calculated 7 molecular descriptors in their study: metal electronegativity (χ), the sum of metal electronegativity for individual metal oxide (∑χ), the sum of metal electronegativity for individual metal oxide divided by the number of oxygen atoms present in a particular metal oxide (∑χ/nO), *N*_Metal_, *N*_Oxygen_, the charge of the metal cation corresponding to a given oxide (χ_ox_), and molecular weight (MW). Two studies [47,50] provided two-dimensional structural information of the ENMs in the form of SMILES (Simplified Molecular Input Line Entry System). Information on the ENM size and aggregation size can also be found in Sizochenko’s study [48]. In addition, 12 electronic descriptors were provided (structural parameters of the ENMs were given by Puzyn et al. [17]), including the standard heat of formation of the oxide cluster (HoF), the total energy of the oxide cluster (TE), electronic energy of the oxide cluster (EE), core-core repulsion energy of the oxide cluster (Core), the area of the oxide cluster calculated based on COSMO (CA), the volume of the oxide cluster calculated based on COSMO (CV), the energy of the highest occupier molecular orbital (HOMO) of the oxide cluster, the energy of the lowest unoccupied molecular orbital (LUMO) of the oxide cluster, the energy difference between HOMO and LUMO energies (GAP), enthalpy of detachment of metal cations Me^n+^ from the cluster surface (Δ*H*_Clust_), enthalpy of formation of a gaseous cation (Δ*H*_Me+_), and the lattice energy of the oxide (Δ*H*_L_). Mu et al. [43] also presented data of 26 computational descriptors for this dataset, detailed information can be found in the supplemental information of the original publication.

Using the same types of 17 ENMs as in Puzyn’s study [17], Pathakoti et al. [46] examined the nanotoxicity to the *E. coli* (Migula) Castellani & Chalmers (ATCC#25254) strain under dark conditions and sunlight exposure for 30 min. Toxicity of ENMs was expressed by the logarithmic values of LC50 in the original article. Information was provided regarding the ENM size (by suppliers), TEM (transmission electron microscopy) particle size, hydrodynamic size, the zeta potential in water, and in KCl solution, and surface area. Moreover, 6 electronic descriptors for metal oxides and 3 for metal atoms were calculated: the larger (less negative) of the HOMO energies of the alpha spin and beta spin orbitals (HHOMO), the alpha and beta LUMO energies (LUMOA and LUMOB, respectively), the absolute electronegativity of the metal oxide calculated from HHOMO and LUMOA (LZELEHHO), the average of LUMOA and LUMOB (ALZLUMO), the molar heat capacity of the metal oxide at 298.15 K (*Cp*), the alpha HOMO and LUMO energies of metal atoms (MHOMOA and MLUMOA, respectively), and the absolute electronegativity of the metal atom calculated from MHOMOA and MLUMOA (QMELECT).

## 4. Existing Nano-(Q)SARs

Suitable modeling tools are capable of extracting meaningful relationships between the nano-structures and nanotoxicity, thus yielding predictive models. The developed nano-(Q)SARs for metallic ENMs are presented in this part. Datasets used for the nano-(Q)SARs are described above in Table 2. Descriptors used in the developed models or identified factors by relevant studies are summarized in Table 6 for further discussion.

### 4.1. Linear Regression Models

Different in silico models predicting the cellular uptake of ENMs by distinct cell lines were developed. In Epa’ study [32], linear models have been reparameterized for the cell uptake of 108 ENMs (87 in training set, 21 in test set) in PaCa2 and HUVEC cells [53]. A method called multiple linear regression with expectation maximization (MLREM) sparse feature reduction was employed to optimize the descriptor set from a pool of 691 descriptors. DRAGON (v5.5), ADRIANA (v2.2), and an in-house modeling software package were used for descriptor calculation. The best performing models used 19 descriptors for PaCa2 cells (*R*^2^_training_ = 0.76, *R*^2^_test_ = 0.79, *SEE* = 0.19, *SEP* = 0.24) and 11 for HUVEC cells (*R*^2^_training_ = 0.74, *R*^2^_test_ = 0.63, *SEE* = 0.34, *SEP* = 0.36).

A partial least squares (PLS) model predicting the cellular uptake (log_10_[ENM]/cell pM) of 109 magnetofluorescent ENMs in PaCa2 cells [53] was constructed by Kar et al. [36]. In this study, a set of 307 descriptors was calculated using the Cerius 2 (v4.10), DRAGON (v6), and PaDEL-Descriptor (v2.11), which was afterwards filtered by the genetic function approximation (GFA). Finally, six molecular descriptors appeared in the developed model:

$$\begin{array}{cc} log_{10}\left[NP\right]/cell\; & =\;3.335+(0.774\times <1-Atype-N-66>)\\ & -\left(0.222\times Atype-N-67\right)+\left(7.360\times <0.600-\sum^{\text{​}}\beta^{\prime }>\right)\\ & -\left(0.101\times Jurs-RPCS\right)-\left(0.00002\times Wap\right)-\left(0.462\times nRNO2\right) \end{array}$$

$$n_{\mathrm{training}}\;=\;89,\;LV\;=\;5,\;R^{2}\;=\;0.806,\;Q^{2}{}_{LOO}\;=\;0.758,\;{Q^{2}}_{Leave-10\%-out}\;=\;0.634,\;{Q^{2}}_{Leave-25\%-out}\;=\;\;0.648,\;SEE\;=\;0.20,\;\bar{r^{2}{}_{m\left(LOO\right)Scaled}}\;=\;0.665,\;∆{r^{2}}_{m\left(LOO\right)Scaled}\;=\;0.113,\;n_{\mathrm{test}}\;=\;20,\;Q^{2}{}_{F1}\;=\;R^{2}{}_{pred}\;\;=\;0.879,\;SEP\;=\;0.12,$$

$$Q^{2}{}_{F2}\;=\;0.868,\;\bar{r^{2}{}_{m\left(test\right)Scaled}}\;=\;0.793,\;{{∆r}^{2}}_{m\left(test\right)Scaled}\;=\;0.115,$$

$$\bar{r^{2}{}_{m\left(overall\right)Scaled}}\;=\;0.679,\;{{∆r}^{2}}_{m\left(overall\right)Scaled}\;=\;0.116.$$

In the model, the descriptors *Atype*-N-66 and *Atype*-N-67 are the hydrophobicity of the N atom in respectively a primary and a secondary aliphatic amine (Al-NH_2_ and Al_2_-NH, respectively), ∑β′ characterizes the measure of electronic features of the molecule relative to molecular size, *Jurs*-*RPCS* stands for the relative positive charge surface area, *Wap* represents for the all-path Wiener index, and *nRNO*2 is the number of aliphatic nitro groups. The leverage and distance to model in X-space (DModX) approaches [58,59] was applied to check the model’s domain of applicability.

Using the same data from Weissleder et al. [53], Ghorbanzadeh et al. [35] proposed a predictive model of cellular uptake (log_10_[ENM]/cell pM) on the basis of a multilayered perceptron neural network technique. A self-organizing map (SOM) strategy was employed combined with stepwise MLR to promote the feature reduction. This procedure provided six most informative descriptors, namely, the number of donor atoms (N and O) for H-bonds (nHDon), the Geary autocorrelation of lag 1 weighted by van der Waals volume (GATS1v), 3D-MoRSE-signal 29/unweighted (Mor29u), D total accessibility index/weighted by Sanderson electronegativity (De), 3D-MoRSE-signal 14/unweighted (Mor14u), as well as the mean electrotopological state (Ms). The linear model has the following form:

$$\frac{log_{10}\left[NP\right]}{cell}=\;2.970-0.130\times \mathrm{nHDon}+0.412\times \mathrm{GATS}1v-0.398\times \mathrm{Mor}29u+1.243\;\times \mathrm{De}-0.163\times \mathrm{Mor}14u+0.045\times \mathrm{Ms}.$$

The model gave a correlation coefficient (*R*) of 0.782 for the training set (RMSE = 0.369) and 0.755 for the prediction (RMSE = 0.357). Williams plot was subsequently put into use for visualizing the domain of model’s applicability.

Nano-(Q)SARs were also derived for the prediction of the cytotoxicity of metallic ENMs. Based on the apoptosis assay of smooth muscle cells from Shaw et al. [54], Epa et al. [32] developed a model consisting of three descriptors for the core material (*I*_Fe3O4_), surface coating (*I*_dextran_), and surface charge (*I*_surf.chg_) of ENMs. The descriptors are considered to have a value of 1 when the condition is present, and 0 when the condition is absent. For instance, *I*_Fe3O4_ is set to be 1 for the ENM with Fe_2_O_3_ core, and 0 when the ENM core is Fe_3_O_4_; *I*_dextran_ is equal to 1 in case of a dextran coating and 0 for others; surface functionality is encoded as 1 (basic), −1 (acidic), or 0 (neutral). Smooth muscle apoptosis was used as the endpoint in the constructed model:

$$SMA\;=\;2.26\left(\;\pm \;0.72\right)-10.73\left(\;\pm \;1.05\right)\times I_{{\mathrm{Fe}}_{2}O_{3}}-5.57\left(\;\pm \;0.98\right)\times I_{\mathrm{dextran}}\;-3.53\left(\;\pm \;0.54\right)\times I_{\mathrm{surf}.\mathrm{chg}}$$

where *n* = 31, *R*^2^_training_ = 0.81, *R*^2^_test_ = 0.86; *SEE* = 3.6, *SEP* = 3.3.

Papa et al. [45] reported three MLR models predicting the potential of ZnO and TiO_2_ ENMs inducing the release of LDH in rat lung cells. Data was retrieved from the study by Sayes and Ivanov [55], which provided values of five descriptors including the engineered size (X0), the size in water (X1), the size in phosphate buffered saline (X2), the concentration (X4), and the zeta potential (X5). The first linear model combined information on both TiO_2_ and ZnO ENMs (all together 31 ENMs):

$${\mathrm{LDH}}_{\left({\mathrm{TiO}}_{2}+\mathrm{ZnO}\right)}\;=\;0.66+0.003X4+0.005X0-4.46E-5X2$$

*R*^2^ = 0.82, *Q*^2^_loo_ = 0.76, *Q*^2^_lmo30%_ = 0.74, *r*^2^_YS_ = 0.10, *s* = 0.11, *F* = 40. The Williams plot for applicability domain of the model was depicted in the original publication. In addition, linear models were also built separately for TiO_2_ (22 ENMs) and ZnO ENMs (15 ENMs):

$${\mathrm{LDH}}_{\left({\mathrm{TiO}}_{2}\right)}\;=\;0.599+0.003X4+0.004X0.$$

*R*^2^ = 0.84, *Q*^2^_loo_ = 0.79, *Q*^2^_lmo30%_ = 0.78, *r*^2^_YS_ = 0.10, *s* = 0.12, *F* = 48.

$${\mathrm{LDH}}_{\left(\mathrm{ZnO}\right)}\;=\;1.041+0.001X1-0.001X2+0.001X4.$$

*R*^2^ = 0.91, *Q*^2^_loo_ = 0.80, *Q*^2^_lmo30%_ = 0.76, *r*^2^_YS_ = 0.22, *s* = 0.08, *F* = 35.

Another approach explicitly and completely based on MLR was reported by Gajewicz et al. [34]. In this case, the cytotoxicity of 18 metal oxide ENMs to the HaCaT cell line was modeled. A set of 27 descriptors was calculated, including 16 quantum-mechanical descriptors and 11 image descriptors derived from Transmission Electron Microscopy (TEM) images. For calculating the quantum-mechanical descriptors, the molecular geometry was optimized at the level of the semi-empirical PM6 method [60] encoded in MOPAC 2009 [61]. Information on the size, size distribution, shape, porosity, and surface area of ENMs was extracted based on TEM images to generate the 11 image descriptors. Two descriptors were afterwards selected by the genetic algorithm (GA), i.e., ∆*H*_f_^c^ and χ^c^. The model can be expressed as follows:

$$\log{\left(LC_{50}\right)}^{-1}\;=\;2.47\left(\;\pm \;0.05\right)+0.24\left(\;\pm \;0.05\right)\times ∆H_{f}{}^{c}+0.39\left(\;\pm \;0.05\right)\times \chi^{c}$$

*F* = 44.6, *p* = *lx*10^−4^, *n* = 18, *R*^2^ = 0.93, *RMSE_C_* = 0.12, *Q*^2^*_CV_* = 0.86, *RMSE_CV_* = 0.16, *Q*^2^*_EXT_* = 0.83, *RMSE_P_* = 0.13

where ∆*H_f_^c^* is the enthalpy of formation of metal oxide nanocluster representing a fragment of the surface, and χ*^c^* represents the Mulliken’s electronegativity of the cluster. The domain of applicability of the model was described by means of a Williams plot.

Using the dataset reported by Gajewicz et al. [34], Pan et al. [44] developed two predictive models incorporating the so-called Improved SMILES-Based Optimal Descriptors. The models predicting the cytotoxicity of metal oxide ENMs to HaCaT cells have the following forms:

$$\log\left(\frac{1}{{\mathrm{LC}}_{50}}\right)\;=\;-0.2909(\;\pm \;0.0664)+0.1038(\;\pm \;0.0027)\times \mathrm{DCW}\left(1,3\right).$$

*n* = 13, *R*^2^ = 0.9606, *Q*^2^_LMO_ = 0.9393, *s* = 0.008, *F* = 268, *p* < 0.0001.

$$\log\left(\frac{1}{{\mathrm{LC}}_{50}}\right)\;=\;0.0012(\;\pm \;0.0048)+0.0778(\;\pm \;0.0001)\times \mathrm{DCW}\left(1,3\right).$$

*n* = 12, *R*^2^ = 0.9997, *Q*^2^_LMO_ = 0.9996, *s* = 0.007, *F* = 1273, *p* < 0.0001.

The number 1 in DCW(1,3) is the coefficient for classification of features into two classes (noise and active); the number 3 in DCW(1,3) is the number of epochs of the Monte Carlo optimization. The characteristics of ENMs involved in these models include molecular weight, cationic charge, mass percentage of metal elements, individual size, and the aggregation size of ENMs.

In addition, in the study by Liu et al. [41] a linear regression model was developed for 24 metal oxide ENMs based on a recently reported dataset [52]. Three descriptors were involved in this model, namely, *E*_c_, ∆*H*_IE_, and χ_MeO_. The model was reported to give an accuracy of 89% for the samples.

The toxicity of metallic ENMs to *E. coli* were also modeled based on laboratory-derived data. Puzyn et al. [17] originally built a dataset for the toxicity of 17 metal oxide ENMs to *E. coli*. Based on the data, a simple and statistically significant nano-QSAR model that used a single descriptor ∆*H*_Me+_ was obtained:

$$\log\left(1/EC_{50}\right)\;=\;2.59-0.50\times ∆H_{\mathrm{Me}+}.$$

*R*^2^ = 0.85, *RMSE_C_* = 0.20, *Q*^2^*_CV_* = 0.77, *RMSE_CV_* = 0.24, *Q*^2^*_EXT_* = 0.83, *RMSE_P_* = 0.19.

Calculation of a pool of 12 variables (Table 5) was executed using the PM6 method as implemented in MOPAC 2009. GA was applied for selecting the most informative descriptors. PLS Toolbox and the Statistics Toolbox for MATLAB were utilized for model development. The leverage approach and Williams plot were employed to visualize the model applicability domain.

Working on the same dataset from Puzyn et al. [17], Kar et al. [37] built a stepwise MLR model as well as a PLS model. Seven descriptors were used for model construction, namely, χ, Σχ, Σχ/*nO*, *N*_Metal_, *N*_Oxygen_, χ*_ox_*, and MW (Table 5). For the MLR model, feature reduction was accomplished by the “stepping criteria” (*F*), and only the descriptor χ*_ox_* was seen in the model:

$$\log\left(\frac{1}{EC_{50}}\right)=\;4.781-\left(1.380\times \chi_{ox}\right).$$

*n* = 17, *R*^2^ = 0.84, *R*^2^*_adj_* = 0.83, *Q*^2^*_LOO_* = 0.81, *Q*^2^*_L_*_-10percent-OUT_ = 0.82;

*Q*^2^*_L_*_-20percent-OUT_ = 0.83, *Q*^2^*_L_*_-25percent-OUT_ = 0.80, ^c^R^2^*_P_* = 0.82.

Meanwhile, the developed PLS model contained two descriptors χ*_ox_* and χ, and has the following form:

$$\log\left(\frac{1}{EC_{50}}\right)=\;4.401-\left(1.324\times \chi_{ox}\right)+\left(0.176\times \chi \right).$$

*n* = 17, *LV* = 1, *R*^2^ = 0.82, *Q*^2^*_LOO_* = 0.75, *Q*^2^*_L_*_-10percent-OUT_ = 0.76;

*Q*^2^*_L_*_-20percent-OUT_ = 0.74, *Q*^2^*_L_*_-25percent-OUT_ = 0.76, ^c^R^2^*_P_* = 0.79.

Characterization of the applicability domain of the model was performed by the leverage approach [58].

Mu et al. [43] also reported MLR models building on the data from Puzyn et al. [17]. Calculation of descriptor was performed using PM6 methods within MOPAC 2012 software package. Pearson and pair-wise correlations, as well as clustering and principal component analysis, were incorporated to obtain optimal structure descriptors for modeling. Among the developed models, a simple but statistically significant nano-QSAR has the following form:

$$\begin{array}{cc} \log\left(\frac{1}{{\mathrm{EC}}_{50}}\right)\;=\; & (4.412\;\pm \;0.165)+\frac{\left(-0.121\;\pm \;0.068\right)Z}{r}\\ & +\left(-0.001\;\pm \;2.57\times 10^{-4}\right)∆H_{\mathrm{Me}+}. \end{array}$$

where *Z* is the ionic charge, and *r* is the Pauling ionic radius. Statistical indicators of the model are as follows: *R*^2^ = 0.8793, *RMSE* = 0.442, *F* = 55.654, *p* = 4.23 × 10^−7^. Leverage approach and Williams plots were used for the characterization of the model applicability domain. Based on the developed model, toxic potencies of other 35 metal oxide ENMs were predicted and visualized in a periodic table. Other models using different descriptors were also described in the study.

Pan et al. [44] also built in silico models using data from Puzyn et al. [17]. The reported models on the basis of the Improved SMILES-Based Optimal Descriptors can be expressed as follows:

$$\log\left(\frac{1}{{\mathrm{LC}}_{50}}\right)\;=\;0.0321(\;\pm \;0.1443)+0.2658(\;\pm \;0.0141)\times \mathrm{DCW}\left(6,11\right).$$

*n* = 10, *R*^2^ = 0.8891, *Q*^2^_LMO_ = 0.8378, *s* = 0.179, *F* = 164, *p* < 0.0001.

$$\log\left(\frac{1}{{\mathrm{LC}}_{50}}\right)\;=\;-0.0076(\;\pm \;0.0306)+0.1420(\;\pm \;0.0020)\times \mathrm{DCW}\left(6,17\right).$$

*n* = 9, *R*^2^ = 0.9824, *Q*^2^_LMO_ = 0.9745, *s* = 0.007, *F* = 391, *p* < 0.0001. The characteristics of ENMs involved in these models include molecular weight, cationic charge, the mass percentage of metal elements, individual size, and the aggregation size of ENMs.

### 4.2. Logistic Regression Models

Liu et al. [39] constructed logistic regression models to classify the effect of nine metal oxide ENMs to BEAS-2B cells into toxic (T) or nontoxic (N). The model with the best classification performance is as follows:

$$\ln\left(\frac{P\left(N\;P\in T\right)}{P\left(N\;P\in N\right)}\right)\;=\;3600.6+103.5\times d+9.5\times \theta_{v}+97.6\times P_{\mathrm{Me}}-58.5\times E_{\mathrm{MeO}}$$

where P(N P∈T) and P(N P∈N) are the probabilities of an ENM being classified as toxic or nontoxic, respectively. *d* is the size of ENM; $\theta_{v}$ is the volume concentration derived from the mass concentration of ENMs; $P_{\mathrm{Me}}$ is the period of the ENM metal in the periodic table; $E_{\mathrm{MeO}}$ is the atomization energy of the metal oxide. The model applicability domain was depicted by principal component analysis.

Liu et al. [41] developed two nano-SAR models based on the logistic regression and quadratic logistic regression methods, respectively. The dataset of Zhang et al. [52] was chosen. This dataset covered data on the toxicity of 24 metal oxide ENMs to BEAS-2B and RAW264.7 cell lines as described above. The quadratic logistic regression model was shown to achieve an accuracy of 89.97% with only two descriptors *E*_C_ and *Z*^2^/*r*. Meanwhile, a marginally better predictability of 90.09% for the logistic regression model was obtained. The molecular descriptors that were included in the logistic regression model are *E*_C_, *E*_Amz_, and *d*.

Logistic regression models were also built by Liu et al. [40] based on an integration of multiparametric bioactivity assays of 44 iron oxide ENMs [54]. The conception of “hit” (significant bioactivity, Signal-to-Noise Ratio > 1.645) was utilized in the study, and the number of hits served as the bioactivity class definition (identifying an ENM as bioactive or inactive) enabling nano-SAR development. Clustering analysis via SOM was also considered besides the number of hits as an alternative to define a class. ENM descriptors included the primary size, zeta potential, R1, and R2. Results showed that the logistic regression model based on class definition of H5 (five hits) possesses the best predictability of 79.3%, using ENM size and R2 as descriptors. The class definition H6 also enabled the construction of a simple logistic regression model (R1 as the sole descriptor) with 78.2% accuracy.

### 4.3. Support Vector Machine Models

A SVM classification model was developed by Fourches et al. [33] using the experimental data of 44 ENMs from Shaw et al. [54]. ENM size, R1, R2, and zeta potential were used as input descriptors, and an arbitrary threshold at Z_mean_ = −0.40 was applied to enable a binary classification. Three clusters of ENMs were identified after assigning a hierarchical clustering procedure. It was found that all monocrystalline iron oxide ENMs were in Cluster II, and all quantum dots appeared in Cluster I. Results of classification confirmed the good predictability of the clustering-based nano-SARs (5-fold external cross-validation) in Cluster II:

Cluster I: *n* = 13, *sensitivity* = 0.5, *specificity* = 0.8;
Cluster II: *n* = 18, *sensitivity* = 0.78, *specificity* = 0.78;
Cluster III: *n* = 13, *sensitivity* = 0.7, *specificity* = 0.4,

where sensitivity = (number of true positives)/(total number of true positives), and specificity = (number of true negatives)/(total number of true negatives) for the binary classification problems.

Another SVM nano-SAR classifying 23 metal oxide ENMs as toxic or nontoxic was built by Liu et al. [41], based on measured toxicological responses in BEAS-2B cells and murine myeloid RAW 264.7 cells following an established protocol [52]. A SOM-based consensus clustering was employed and afterwards identified three ENM clusters. Clusters II and III contained ENMs being reported as toxic, and were thus grouped into a single cluster of ENMs classified as having a positive response. ENMs in Cluster I were labeled as nontoxic. A pool of 30 descriptors were initially considered including information on the fundamental metal oxide, energies or enthalpies of metal oxide, ENM size, zeta potential and isoelectric point, and ENM energy. Descriptor selection was accomplished by the evaluation of models derived from all possible descriptor combinations. The SVM algorithm successfully correlated the cytotoxicity of ENMs with ENM conduction band energy (*E*_C_) and ionic index of metal cation (*Z*^2^/*r*). The penalty factor and the kernel width of the SVM model were determined to be 128 and 2, respectively. The discriminant function of the SVM model was given by

$$f\left(x\right)\;=\;\overset{6}{\underset{i\;=\;1}{\sum }}\alpha_{i}e^{-2[{\left(x_{i,1}-x_{1}\right)}^{2}+\left(x_{i,2}-x_{2}{)}^{2}\right]}+b$$

where **x** refers to the ENM identified by the normalized descriptors vector [*Z*^2^/*r*, *E*_C_] (i.e., *x*_1_, *x*_2_), and *x_i_*_,1_ and *x_i_*_,2_ stand for the normalized first and second descriptors identified as support vectors. The values of α*_i_* (*i* = 1–6) were represented by ZnO (82.342), Ni_2_O_3_ (128), Mn_2_O_3_ (83.696), NiO (−70.471), CeO_2_ (−95.566), and Fe_2_O_3_ (128) with *b* being −10.888. The model was reported to give a predictive accuracy (obtained via 0.632 estimator) of 93.74%. The model applicability domain was characterized by a probabilistic approach [62].

### 4.4. Artificial Neural Network Models

Based on the experimental results of Shaw et al. [54], a Bayesian regularized ANN model was constructed predicting the smooth muscle cells’ apoptosis triggered by 31 ENMs [32]. Model statistics are as follows: *n* = 31, *R*^2^_training_ = 0.80, *R*^2^_test_ = 0.90, *SEE* = 2.8, *SEP* = 2.9. Meanwhile, an ANN nano-SAR was also built in the study, modeling the cellular uptake in HUVEC and PaCa2 cells [53]:

Cellular uptake in HUVEC cells: *R*^2^_training_ = 0.70, *SEE* = 0.30, *R*^2^_test_ = 0.66, *SEP* = 0.33, descriptor number = 11;

Cellular uptake in PaCa2 cells: *R*^2^_training_ = 0.77, *SEE* = 0.15, *R*^2^_test_ = 0.54, *SEP* = 0.28, descriptor number = 19.

Besides the above-mentioned MLR model developed by Ghorbanzadeh et al. [35], another nano-SAR on the basis of a multilayered perceptron neural network technique was also introduced in their study. The SOM strategy combined with a stepwise MLR selected six most informative descriptors, namely, nHDon, GATS1v, Mor29u, De, Mor14u, and Ms. The derived model gave a performance in terms of values of R^2^ of 0.934 for the training set, 0.945 for the internal test set, and 0.943 for the external test set. The calculated RMSE values are 0.146, 0.121, and 0.214 for respective training, internal test, and external test sets, while the corresponding values of *F* are 531, 142, and 65, respectively. The applicability domain of the model was firstly evaluated by the approach based on ranges of individual descriptors. A Williams plot was subsequently put into use for visualizing the domain of applicability.

### 4.5. k-Nearest Neighbor Models

A classification model employing the *k*NN approach was developed in the study by Fourches et al. [33]. The model was proposed to predict the cellular uptakes of 109 ENMs in PaCa2 cells [53]. Coefficients of correlation *R*_abs_^2^ were shown to range from 0.65 to 0.80 for the external sets, and from 0.67 to 0.90 taking into account the applicability domain which was defined by the Euclidean distance approach. In this study, descriptors that most frequently occurred in the models (1-5-fold cross-validations) with the highest prediction accuracy were identified. The top 10 descriptors ranked by averaged frequency were reported to be SlogP_VSA1, SlogP_VSA2, SlogP_VSA5, b_double, SlogP_VSA0, PEOE_VSA+1, vsa_don, vsa_other, vsa_base, and PEOE_VSA_FPOS. The SlogP_VSA0 and SlogP_VSA1, along with other descriptors with relatively low frequency such as GCUT_SLOGP_0 and BCUT_SLOGP_0, are considered to be generally related to the lipophilicity. For instance, the PaCa2 uptake of ENMs was observed to be positively correlated with the enrichment of lipophilic compounds on ENM surfaces (value of GCUT_SLOP_0). Other discriminated factors affecting the PaCa2 uptake of ENMs were found to be about the molecular refractivity, the specific van der Waals surface area, and the electrostatic properties. The applicability domain of the model was characterized by the Euclidean distance.

An attempt of predicting the cytotoxicity of 44 iron oxide ENMs based on *k*NN was also reported by Liu et al. [40]. As described above, different numbers of hits were discussed in the study for introducing class definitions besides the clustering analysis via SOM. The results showed that a *k*NN model using SOM-based consensus clustering gave the best predictive performance of 74.9% accuracy. Three descriptors, ENM size, R1, and R2, were obtained in this model. Meanwhile, H4 class definition was also deemed to be a suitable choice, which enabled the development of a *k*NN model correctly predicting 74.3% of the samples.

### 4.6. Other Models

Chau and Yap [30] attempted to correlate the cellular uptake in PaCa2 with the calculated parameters from PaDEL-Descriptor (v2.8). By lowering the threshold value of significant uptake into PaCa2, 56 ENMs with a cellular uptake of more than 5000 ENMs per cell [53] were considered as a positive class, and the other 49 were defined as the negative class. Based on the four modeling techniques of naive Bayesian classifier (NBC), logistic regression, *k*NN, and SVM, 2100 candidate models were developed while only 102 of them were qualified according to the selection criteria. To build a final consensus nano-SAR model, the top 5 candidate models were chosen consisting of 3 *k*NN, 1 SVM, and 1 NB models. The consensus model gave a good predictive performance with sensitivity of 98.2% and specificity of 76.6% for the dataset. Descriptors that commonly appeared in the candidate models include the number of CH_2_ groups, primary, secondary, and tertiary nitrogens, halogens (fluorine, bromine, iodine), sulfur atoms, fused rings, and hydrogen bonding. Most of the descriptors that contributed to the model were interpreted as related to the lipophilicity (e.g., number of lipophilic groups). Other factors such as the hydrogen bonding between nitrogen and hydrogen, and the sulfur and various halogen atoms were also found to affect the cellular uptake of ENMs. This is in agreement with the study by Fourches et al. [33].

Chen et al. [31] reported several nano-SARs for the categorization of ENM hazards to different biota. The toxicity data was retrieved from the database of Chen et al. [63] and the online chemical modeling environment platform [64]. Functional tree, C4.5 decision tree, random tree, and Simple CART approaches were employed for model development. Global nano-SARs across species using LC50 data were shown to correctly predict more than 70% of the samples in training (320 ENMs) and test sets (80 ENMs) based on the functional tree, C4.5 decision tree, and random tree methods. The species-specific nano-SARs were also derived for *Danio rerio*, *Daphnia magna*, *Pseudokirchneriella subcapitata*, and *Staphylococcus aureus* with good predictivity. Summarized from the developed models, the molecular polarizability, accessible surface area, and solubility were identified as key factors affecting the biological activities of metallic ENMs.

Moreover, Zhang et al. [52] reported a regression tree model using the metal dissolution of metal oxide ENMs and energy of conduction band to predict the toxicity potential of 24 metal oxide ENMs. With the data from Zhang et al. [52], Sizochenko et al. [49] developed causal inference nano-SARs for BEAS-2B and RAW 264.7 cell lines (24 metal oxide ENMs for each cell line) with high predictivity. Luan et al. [42] and Kleandrova et al. [38] developed the novel QSTR-perturbation (quantitative structure-toxicity relationship) models assessing the cytotoxicity and ecotoxicity of various types of ENMs. The factors of molar volume, polarizability, size of ENMs, electronegativity, and the hydrophobicity and polar surface area of surface coatings were indicated by the reported models. Singh and Gupta [47] previously performed three cases of nano-(Q)SAR study for metallic ENMs on the basis of the datasets generated by Puzyn et al. [17], Shaw et al. [54], and Weissleder et al. [53]. In their study, classification and regression models were constructed predicting various biological effects of the ENMs by an ensemble learning based strategy known as stochastic gradient boosting and bagging algorithms. Results showed that the developed models are of robustness, and no over-fitting of data was present in any case study. Furthermore, attempts to link the information of ENM structures to corresponding biological effects were also made using other modeling techniques, such as the Monte Carlo method [50,51], NBC and linear discriminate analysis [40,41], random forest regression [48,49], and the self-written least-squares fitting program [46].

## 5. Interpret Mechanisms of ENM Biological Activities with Developed Models

To enable the fast and inexpensive high-throughput prediction of diverse biological effects caused by ENMs, reliable nano-(Q)SARs should be based on mechanistic knowledge [28]. Only when information on the underlying mechanisms is incorporated in modeling, proper and reliable extrapolation towards untested ENMs or organisms can be performed. Based on existing experimental data related to the cellular uptake of ENMs as well as the toxicity of ENMs to different cell lines and to *E. coli*, various nano-(Q)SARs were developed (Table 1). The significant descriptors introduced in the aforementioned nano-(Q)SAR studies were shown to be able to provide vital structural information on the factors affecting ENMs’ cellular uptake and toxicity. Therefore, information on these descriptors as summarized in Table 6 is linked to the current understanding of the mechanisms of nanotoxicity.

### 5.1. Cellular Uptake of ENMs

**Figure 1:**
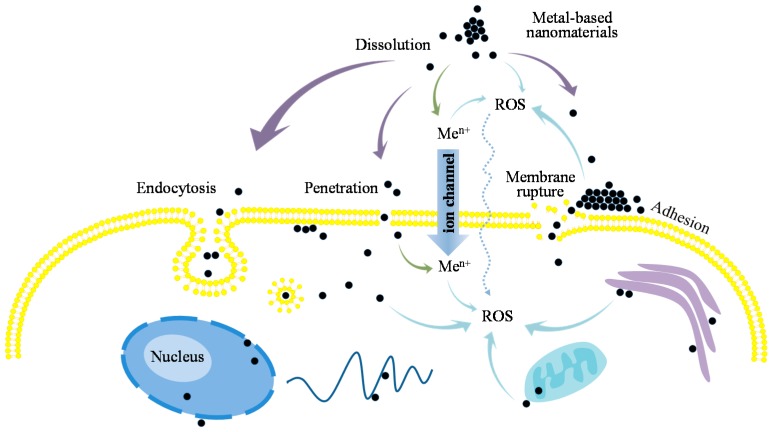
Overview of hypotheses associated with the responses of cellular membrane to the introduction of ENMs. It is assumed that endocytosis, penetration, adhesion of ENMs upon the cellular membrane, and cellular membrane rupture could possibly occur. Cellular membrane rupture is also considered to lead to the internalization of ENMs via the damage sites. Scenario of relevant ion release from ENMs, generation of reactive oxygen species (ROS), and ENMs-contacted interactions are also depicted.

Once entering into the medium, ENMs may undergo various extra-and intracellular physical-chemical reactions such as dissolution, ion release, reactive oxygen species (ROS) generation, interaction with subcellular structures (e.g., cellular membrane, mitochondrion), and internalization into the cells (Figure 1). Cellular uptake of ENMs is always seen as an important process of ENMs’ internalization and subsequently initiating the ENM contact-mediated or dissolved ion-associated intracellular reactions. As hypothesized, ENMs are conventionally transported into cells through endocytosis, a form of active transport in which cells take in ENMs by engulfing them [65]. Possible endocytotic processes proposed include phagocytosis, macropinocytosis, caveolae-dependent and clathrin-mediated endocytosis, and non-clathrin-, non-caveolae-mediated endocytosis [65,66]. In addition, other responses of cellular membranes to adsorption of ENMs were also shown to exist. On the basis of a dissipative particle dynamics simulation study, Yue and Zhang [67] concluded that surface adhesion, membrane penetration, and even ENM-induced membrane rupture could occur upon the ENM attachment to cellular membranes. Lin et al. [68] and Xia et al. [69] also demonstrated that ENMs could access the cellular interior through direct membrane penetration.

In these internalization processes, the surface properties of ENMs are essential for ENM-biomolecule interactions and are deemed to be able to alter cellular uptake pathways. In the experiment of Weissleder et al. [53], a diversity of cellular uptake processes was especially observed for the PaCa2 cells. These authors consequently concluded that the translocation process is highly dependent on the surface modification of the ENMs. The studies showed that the lipophilicity of the surface molecules is an important discriminating factor that determines the chemical ability to interact with the lipid core of membranes [70]. Fourches et al. [33] reported that four descriptors (out of the top ten with the highest averaged frequency) SlogP_VSA0, SlogP_VSA1, SlogP_VSA2, and SlogP_VSA5 are intimately correlated with the molecular lipophilicity of surface compounds. The ENMs with a higher PaCa2 cellular uptake are generally highly enriched for lipophilic surface modification (higher descriptor value). This is consistent with the results of Epa et al. [32] in which C-005 (associated with hydrophobicity) was observed as a factor affecting ENMs’ cellular uptake. Further confirmation was obtained by the appearance of *Atype*-N-66 and *Atype*-N-67 (Kar et al., 2014a) in a PLS model, and the number of lipophilic groups (CH_2_, fused rings) in the consensus model of Chau and Yap [30]. Additionally, the hydrogen bonding capacity of surface modifiers was explained to be one of the driving factors of ENMs’ membrane penetrability [30]. An ANN model predicting cellular uptake of ENMs by HUVEC was reported to include the descriptors nRCONHR and nArOCON, which characterize molecular hydrogen bonding capacity [32]. In the same study, nN, nArOH, H-053, and O-058 were also found in the MLR model and were likewise interpreted as affecting the capability of H-bonding. In other nano-(Q)SARs, descriptors considered to correlate with this factor include nHDon [35], WPSA-2, nHBDon, and nHBAcc [47]. Hence, these informative descriptors found in the developed nano-(Q)SARs corroborated previous experimental observations, confirming that the lipophilicity of surface compounds is of significant importance for the cellular uptake of ENMs.

Additionally, shape, size, and flexibility of the surface compounds also play an important role in determining ENMs’ passive transport across biological membranes. For instance, descriptors (not exclusively) characterizing molecular branching, such as nR10, nCIR, nCs [32], *Wap* [36], GATS1v, Mor29u, Mor14u [35], SP-5, VP-4, and VPC-6, have been constantly observed in studies [47]. The Mor29u, Mor14u, SP-5, VP-4, and VPC-6 meanwhile also contain information regarding the molecular three-dimensional structures (e.g., mass, size, flexibility, and overall shape). Other relevant descriptors include ASP, DISPm, QZZm, QYYp, SPAM [32], ∑β' [36], De [35], MOMI-XZ, nRotB [47]. Moreover, impacts on ENMs’ cellular uptake were also reported to derive from the molecular reactive surface and electronegativity. Molecular reactive surface-related descriptors in the nano-(Q)SARs are vsa_don, vsa_other, vsa_base, PEOE_VSA_FPOS, PEOE_VSA + 1 [33], *Jurs*-*RPCS* [36], WNSA-3 [47]. Descriptors associated with molecular electronegativity were observed to be nRNO2 [36], primary, secondary, and tertiary N, and halogens [30]. It is not surprising that these factors of ENM surface modifiers may influence the ENM-biosurface interactions and pose effects on the cellular uptake of ENMs, independently or cooperatively. Either shape or size, or flexibility of surface modifiers of ENMs would affect the interactions between these molecules and the molecular sites of biosurfaces, change the conformation of binding complexes, and ultimately mediate the subsequent ENM-biosurface reactions in which the nature of the reactive surface and electronegativity also play a role.

As seen in Figure 1, internalization of ENMs into cells is generally considered as a crucial biological process triggering nanotoxicity. However, the adsorption of ENMs on cellular membranes may also affect cellular membrane integrity and lead to the formation of defects through the membranes [67,68,69,71]. This could probably result in the direct internalization of ENMs through the damage sites of membranes as well as the release of intracellular components, which causes cell death. Notably, the extracellular release of ions and the formation of ROS are also considered to be factors affecting the toxicity of ENMs in some cases. von Moos and Slaveykova [72] reported that intracellular ROS generation can be stimulated by the presence of extracellular ROS as a response. The released ions and derived ROS may as well interact with cellular membranes, and dependently and/or independently influence ENMs’ cellular uptake. This gives a possible explanation on the presence of the descriptor ionization potential (IP) in the nano-SAR of Singh and Gupta [47], and may also explain why the electronegativity-related descriptors nRNO2 [36], primary, secondary, and tertiary N, and the halogens [30] generally appeared in relevant nano-(Q)SAR studies.

### 5.2. ENMs-Induced Biological Effects

**Figure 2:**
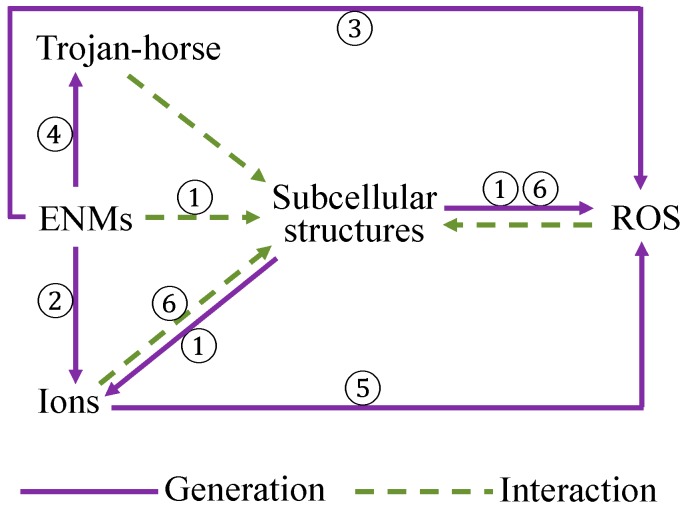
Schematic illustration of possible mechanisms of metallic ENMs triggering nanotoxicity. ① ENMs directly in contact with subcellular structures, which can promote the release of ions and ROS generation; ② ENMs releasing ions; ③ ENM contact-mediated ROS generation; ④ Trojan-horse mechanism triggered by ENMs; ⑤ released ions increasing the formation of ROS; ⑥ ion-dependent interactions that may lead to cellular damage or trigger ROS formation.

It is well-known that ENMs are capable of eliciting adverse biological effects by directly or indirectly triggering a series of physical-chemical reactions and ultimately causing cell damage. Reportedly, toxicity of ENMs can occur via a single mechanism or via combinations of the following mechanisms (Figure 2): (i) direct interactions with subcellular structures or biomolecules (e.g., membranes, mitochondria, proteins, DNA) that can lead to, for instance, mitochondrial damage, the denaturation of proteins, and the formation of corona; (ii) the release of chemical constituents from ENMs such as metal ions; (iii) the surface property-based chemical reactivity of ENMs, e.g., photochemical, catalytic and redox properties; and (iv) Trojan-horse type mechanisms, so-called intruders in which ENMs act as vectors for transporting toxic chemicals [73].

Generally, there is no doubt that metal-ions leaching from ENMs could act as a key factor causing biological effects of ENMs. Once the ENMs release dissolved ions surrounding the cells, it is often difficult to experimentally distinguish the effects caused by conventional metal ion release from the nano-specific effects (i.e., the adverse effects elicited by ENMs by different mechanisms compared to the toxicity triggered by corresponding bulk counterparts, or the toxic effects that can only be observed when the material is in its nano size). In such a context, the toxicity induced by ENMs is always considered to be intimately correlated with ENM dissolution. Comparable results on the toxicity of ZnO ENMs and Zn salts have been observed for the examples of *Pseudokirchneriella subcapitata* [74], *Thamnocephalus platyurus*, and *Daphnia magna* [75,76], and *E. coli* [77]. Result of studies on the toxicity of CuO ENMs to multiple species is also in agreement with this conclusion [75,78]. It is commonly believed that ion release could occur after the cellular internalization of ENMs, which consequently results in different mechanistic pathways of nanotoxicity. For instance, Stohs and Bagchi [79] proposed a Haber-Weiss-Fenon cycle describing the stimulation of ion-leaching to ROS generation, taking Cu^2+^ as an example:

$$O_{2}^{•}{}^{-}+{\mathrm{Cu}}^{2+}\to O_{2}+{\mathrm{Cu}}^{+}$$

$${\mathrm{Cu}}^{+}+H_{2}O_{2}\to {\mathrm{Cu}}^{2+}+{\mathrm{OH}}^{-}+{\mathrm{OH}}^{•}$$

where the ROS such as superoxide anion radicals ($O_{2}^{•}{}^{-}$) could be derived from the one-electron reduction of molecular oxygen O_2_:

$$O_{2}+e\to O_{2}^{•}{}^{-}$$

In the Haber-Weiss-Fenton cycle, Cu^2+^ acts as catalysts of the formation of hydroxyl radicals, which enhances the generation of ROS. Meanwhile, it was suggested that the release of ions could be accompanied by ROS formation as well such as in the Fenton reaction [34]:

$$\mathrm{Fe}+O_{2}+2H^{+}\to {\mathrm{Fe}}^{2+}+H_{2}O_{2}$$

$${\mathrm{Fe}}^{2+}+H_{2}O_{2}\to {\mathrm{Fe}}^{3+}+{\mathrm{OH}}^{•}+{\mathrm{OH}}^{-}$$

Evidence from nano-(Q)SAR studies also demonstrated the contribution of ion release to nanotoxicity. The influence of metal solubility on nanotoxicity was indicted by the developed models [31,52]. Puzyn et al. [17] developed a linear model based on the sole descriptor ∆*H*_Me+_ predicting toxicity of metal oxide ENMs to *E. coli*. It was explained that ∆*H*_Me+_ is an efficient descriptor characterizing the stability of metal oxides, which is associated with both the lattice energy of oxides and the sum of the ionization potentials of a given metal. The release of cations with a smaller charge is seen as more energetically favorable than that with a larger charge [43]. This explains the observations of previous studies giving an order of oxide toxicity as follows: Me^2+^ > Me^3+^ > Me^4+^ [17]. According to Kar et al. [37], the charge of the metal cation corresponding to a given oxide (χ*_ox_*) was also used for the parameterization of nanotoxicity data. In the study by Liu et al. [41], the descriptor ionic index of metal cation *Z*^2^/*r* was involved in building classification nano-SARs. *Z* is the ionic charge and *r* represents the Pauling ionic radius of the released ions [44]. *Z*^2^/*r* is a measure of the involvement of a metal ion into electrostatic interactions, and is able to provide information on the affinity of a metal ion for water molecules. Likewise, such a form of index was also used in random forest models [48,49], coupled with a parameter (*S*_1_) describing the van der Waals interactions between surface molecules or cations. Other descriptors related to the ionic charge and/or radius include polarization force [43], covalent index, the tri-atomic descriptor of atomic charges, and the tetra-atomic descriptor of atomic charges [49].

Accordingly, Gajewicz et al. [34] employed two descriptors, i.e., enthalpy of formation of metal oxide nanocluster (∆*H_f_^c^*) and the Mulliken’s electronegativity of the cluster (χ*^c^*), to linearly explain the cytotoxicity of metal oxide ENMs to HaCaT. The ∆*H_f_^c^* is associated with the energy of a single metal-oxygen bond in oxides (*E*_∆*H*_°), which can be expressed as follows [80]:

$$E_{∆H^{o}}\;=\;\frac{2{∆H}_{f}^{o}\cdot 2.612\times 10^{19}}{N_{A}n_{e}}$$

where *N*_A_ is the Avogadro number, and *n*_e_ is the number of electrons involved in the formation reaction. A high ∆*H*_f_^c^ value indicates a strongly bound cation of large formal charge in the oxides, and thus affects the detachment of metal cation from the surface of the ENMs. As for χ*^c^*, Burello and Worth [29] introduced that the electronegativity value of metal oxide (χ*_oxide_*) can be calculated from that of the corresponding cation (χ*_cation_*) using the following equation [81]:

$$\chi_{oxide}\approx 0.45\chi_{cation}+3.36.$$

Therefore, a higher value of χ*_cation_* indicates a stronger ability of a cation to attract electrons in the Haber-Weiss-Fenton cycle, which in turn results in higher reactivity of the metal oxide ENMs [34]. The two descriptors ∆*H_f_^c^* and χ*^c^* meanwhile also refer to ENMs’ surface redox activity. Burello and Worth [29] reported that the energy of a band gap (*E_g_*) can be obtained based on the *E*_∆*H*_°:

$$E_{g}\;=\;Ae^{0.34E_{∆H^{o}}}$$

and thus the conduction and valence band energies of oxides become

$$E_{c}\;=\;-\chi_{oxide}+0.5E_{g}+E_{shift}$$

$$E_{v}\;=\;-\chi_{oxide}-0.5E_{g}+E_{shift}$$

where *E_shift_* represents the value changes of band edges in respect to the solution’s pH. As hypothesized, the redox potentials of relevant biological reactions could be unbalanced if they lie closer to the *E*_c_ or *E_v_*, thereby causing cellular oxidative stress [52]. This was confirmed by Liu et al. [41] who identified *E*_c_ and χ*_oxide_* for the development of nano-(Q)SAR models, and Kar et al. [37], Kleandrova et al. [38], Luan et al. [42], and Sizochenko et al. [49] who used metal electronegativity as one of the modeling parameters. Pathakoti et al. [46] as well obtained two descriptors (absolute electronegativity of the metal and metal oxide) for describing the toxicity of metal oxide ENMs to *E. coli* under darkness. Other descriptors considered to be associated with the surface redox properties of ENMs and causing oxidative stress include ∆*H*_IE_, *E*_Amz_ [39,41], *CI*, *S*_3_ [48], *Cp*, ALZLUMO [46], and polarizability [31,38,42] in relevant nano-(Q)SAR studies.

On the other hand, other than the general consensus taking ion release and ROS generation as driving factors in nanotoxicity, it is evident that other mechanisms of toxicity also play a vital role in certain cases. Xiao et al. [82] reported that, for both Cu and ZnO ENMs, the particles per se, rather than the dissolved ions, provided the major contribution to the toxicity to *Daphnia magna* (26% and 31%, respectively). Similarly, Hua et al. [83] also revealed a dominant contribution of ZnO ENMs over the Zn ion tested for zebrafish embryos, for which the dissolution-driven mechanism of ENMs toxicity apparently does not apply. More precisely, it was shown that the shape of ENMs significantly affect ENMs’ toxicity, as needle-shaped ZnO ENMs were proven to be the most toxic to *Phaeodactylum tricornutum* as compared to morphologically different ENMs with equal solubility and ion release [84]. Observations of nanotoxicity affected by the shape of ENMs were also reported for ZnO nanospheres, nanosticks, and cuboidal submicron particles [83]. Computational studies proved the involvement of surface property-related descriptors in nano-(Q)SAR modeling, such as the surface area and coating [31,32,47,49], the hydrophobicity and polar surface area of surface molecules [38,42], the surface-area-to-volume ratio [49], zeta potential [45], and the Wigner–Seitz radius of the oxide’s molecule, which describes the available fraction of molecules on the surface of ENMs [48,49]. The Wigner-Seitz radius also relates to the molecular weight and density, and therefore the molecular volume, all of which have been indicated in the models [38,42,44,49]. Descriptors relating to ENM size [38,39,40,41,42,44,45,47], material composition [32,39,44,47,48], and aggregation behaviors [44,45,48,49] were also concluded to affect nanotoxicity [85] from the aspects of relevant computational studies.

As mentioned above, ENMs may induce toxicity by direct steric hindrance or by binding with important reaction sites, or by indirect behaviors such as ion release, ENM surface-contacted interactions, or by acting as carriers for toxic chemicals (as in Figure 2). Take the case of TiO_2_ as a typical example of ENM surface-mediated photochemical reaction, in which detachment of an electron could be activated by solar radiation [37]:

$${\mathrm{TiO}}_{2}\overset{hv}{\Rightarrow }{\mathrm{TiO}}_{2}^{+}+\bar{e}$$

$$\bar{e}+O_{2}\to O_{2}^{•}{}^{-}$$

$$O_{2}^{•}{}^{-}+2H^{+}+\bar{e}\to H_{2}O_{2}$$

$$O_{2}^{•}{}^{-}+H_{2}O_{2}\to {\mathrm{OH}}^{}+{\mathrm{OH}}^{-}+O_{2}$$

$$H^{+}+H_{2}O\Rightarrow {\mathrm{OH}}^{•}+H^{+}$$

The binding of ENMs with organelles could also cause a release of ions from interior storage due to the loss of membrane integrity. Unfried et al. [66] reported that ENMs interacting with mitochondria are able to promote the release of interior-stored Ca^2+^. The released ions are capable of triggering ROS production by direct catalysis, e.g., the Haber-Weiss-Fenton cycle, or indirect interference of biological functions such as interrupting the mitochondrial electron transduction [72]. In addition, the ions *per se* could unbalance intracellular biological functions, eliciting inflammation, lysosomal damage, and inhibiting cellular respirations [86]. The interactions of ENMs with subcellular structures (e.g., membrane-bound enzymes) were also shown to be capable of enhancing ROS production. Interestingly, the presence of extracellular ROS was reported to be able to elevate intracellular ROS generation as depicted in Figure 2 [72].

In summary, the characteristics of ENMs may pose effects on the toxicity of ENMs as related to a single mechanism or to combinations of possible mechanisms. Analysis of the descriptors discussed in existing nano-(Q)SAR studies assists in offering a statistical overview extracted from the complicated mechanistic pathways, and enables a mechanistic interpretation on the basis of the main driving factors. As discussed above, ENMs’ surface properties are vital for their uptake by cells concerning the lipophilicity, hydrogen bonding capacity, electronegativity, shape, size, and flexibility of the surface modifiers. As for ENM-triggered toxicity, properties correlating with the ability of ion release and ROS generation, along with the information about the size, surface redox properties, and composition of ENMs, could be important indicators.

## 6. Conclusions and Outlook

Enabling the development of reliable nano-(Q)SARs is capable of reducing the time and cost needed for conventional experimental evaluations, and thus benefits the risk evaluation and assessment of ENMs for regulatory purposes. Even though the promising potential of extending (Q)SARs into nanotoxicity has been addressed, the nano-(Q)SAR approach is still in its infancy. As far as it is understood, the scarcity of (properly documented) experimental data is regarded as one of the major drawbacks in building nano-(Q)SAR models. The information provided in Table 2 indicated a very limited availability of existing data as only a few datasets constantly appeared in the overview of nano-(Q)SAR studies, in spite of the numerous scientific programs on ENMs’ safety and design. This suggests (i) that most of the studies reported do not meet the modeling criteria, which, amongst others, include a lack of relevant pristine or characterization data, a lack of a description of the method used, or a lack of reporting of a consistent endpoint; or (ii) that the integration of existing experimental data based on various studies is currently lacking, which hinders the inclusion of this valuable information into the nano-modeling field [87]. Therefore, in light of advancing computational nanotoxicology, a summary and organization of potentially useful nanotoxicity is essential. Besides the data quantity, the quality of experiment data collected should also be taken care of for the data-driven nano-(Q)SAR approach, which was found absent in the relevant studies owing to the single-source strategy of retrieving data for a model. It is suggested that the quality of experimental information assembled from various sources ought to be evaluated by suitable tools before model construction. This is seen as helpful for improving the statistical significance and predictability of a model.

Meanwhile, the grouping and characterization of ENMs as well remain crucial for developing nano-(Q)SARs. In general, the strategies of grouping ENMs are considered to be ENM composition-based, toxic mode of action-based, or further clustering-based [33,40]. Characterization of ENMs will subsequently be carried out for the ENM groups in terms of molecular structural descriptors. However, concerns have always been expressed with regard to the question whether it is possible to build nano-(Q)SARs without considering ENMs’ dynamic transformations in the exposure medium. On the one hand, it is well-known that, once entering into a medium, ENMs are more likely to strongly react with the components of the test medium and undergo dramatic changes of surface properties. These surface transformations would in return affect ENMs’ reactivity and subsequent biological behaviors (e.g., cellular uptake, interaction with subcellular structures). In such a context, modeling based solely on the information of ENMs’ pristine structures could be biased and could result in poor predictability and reliability of the models generated. Meanwhile, on the other hand, a few efforts did provide evidence regarding the feasibility of building nano-models using the characteristics of pristine ENMs [17,36,46,47,48]. In fact, the possibility exists that the characteristics of pristine ENMs influence the biological effects of ENMs by affecting ENMs’ dynamic transformations in media, and it can be hypothesized that even though changes of ENM property could occur in the exposure media, the characteristics of the pristine ENMs still are linked to adverse biological effects of ENMs. In this circumstance, constructing nano-(Q)SARs with only the characteristics of pristine ENMs could enable the development of high-throughput protocols for non-testing nanotoxicity evaluation. This is expected to allow for a reduction in the high cost and time needed by conventional evaluation methods. However, all the proposed hypotheses should be further confirmed by more nano-(Q)SAR studies in pace with the advance of computational nanotoxicology. Due to the limitation of literature search based on empirical selection of key words and the limitation of manual supplementation with publications of interest not in the search records, there may be results that are not included and thus not discussed in the review. Evaluating the performance of reported models are also not considered in the review, which may need to be thoroughly discussed as a next step.

In conclusion, the added value of this review can be summarized as follows:

(i) A general overview is provided of the datasets being widely used in nano-(Q)SAR studies, and the characterization of ENMs in these datasets is discussed. Experimental data are shown to be mainly available concerning the cellular uptake by different cell lines (e.g., PaCa2, HUVEC), cytotoxicity to cells (e.g., HaCaT, BEAS-2B), and the toxicity to *E. coli*. A limited usage of existing data in relevant investigations was observed.
(ii) An overview of nano-(Q)SARs so far developed, based on a variety of modeling techniques such as linear and non-linear regressions (MLR, PLS, and logistic regression), SVM, ANN, and *k*NN, is presented.
(iii) An interpretation of the underlying mechanisms of ENM toxicity and cellular uptake is provided on the basis of the descriptors discussed in nano-(Q)SAR studies. The surface properties of ENMs are deemed vital for the uptake of ENMs by different cell lines, such as lipophilicity, hydrogen bonding capacity, electronegativity, shape, and size of surface modifiers. The capability of releasing ions and generating ROS, surface redox properties of ENMs are concluded to be important indicators for evaluating the toxicity of ENMs.
(iv) An outlook is presented regarding the experimental data needed for future modeling and the need of proper characterization of ENMs. Owing to the limited data availability, optimizing the usage of existing information of nanotoxicity should be deliberately considered; integrating relevant available data thus becomes vital for the development of nano-(Q)SARs. Meanwhile, whether or not the dynamic transformations of ENMs in media play a vital role in the computer-aided nanotoxicity also ought to be further discussed.

## Figures and Tables

**Table 1:** Overview of the peer-reviewed literatures on nano-(Q)SARs, as generated by means of an advanced literature search in the Web of Science™ Core Collection on 22 February 2017, and supplemented with a manual collection of relevant publications not included in the search record. Apart from the references obtained, a general description is given for the models reported.

Reference	Brief Description
Cellular uptake	
[30]	Developed a final consensus model based on top 5 candidate models constructed by naive Bayes, logistic regression, *k*-nearest neighbor (*k*NN), and support vector machine (SVM), predicting the cellular uptake of 105 ENMs (single metal core) by PaCa2 pancreatic cancer cells (PaCa2)
[32]	Modeled cellular uptake of 108 ENMs in human umbilical vein endothelial cells (HUVEC) and PaCa2 cells using multiple linear regression (MLR) with the expectation maximization method
[33]	Generated models predicting the cellular uptake of 109 ENMs in PaCa2 cells using the *k*NN method
[35]	Cellular uptake of 109 magnetofluorescent ENMs in PaCa2 cells was modeled using MLR and multilayered perceptron neural network, descriptor selection was performed by combining the self-organizing map and stepwise MLR
[36]	Developed a model establishing the cellular uptakes of 109 magnetofluorescent ENMs in PaCa2 cells
[47]	Predictive models were built based on cellular uptake of 109 ENMs in PaCa2 cells
[51]	Cellular uptake of 109 ENMs with the same core but different surface modifiers in the PaCa2 cells was modeled based on SMILES-based optimal descriptors
Cytotoxicity	
[29]	A model was proposed to show that the oxidative stress potential of metal oxide ENMs could be possibly predicted by looking at their band gap energy
[32]	Modeled cytotoxicity of 31 ENMs to vascular smooth muscle cells based on MLR and Bayesian regularized artificial neural network
[33]	Generated models predicting the cytotoxicity of 44 ENMs with diverse metal cores using the SVM method
[34]	Applied the MLR method combined with a genetic algorithm to describe the toxicity of 18 metal oxide ENMs to the human keratinocyte cell line (HaCaT)
[39]	Classification models (logistic regression) were developed to predict the cytotoxicity of nine ENMs to the transformed bronchial epithelial cells (BEAS-2B)
[40]	A nano-SAR was developed classifying 44 iron-based ENMs into bioactive or inactive, using a naive Bayesian classifier based on 4 descriptors: primary size, spin-lattice, and spin-spin relaxivities, and zeta potential
[41]	SVM nano-SAR model was constructed on basis of the cytotoxicity data of 24 metal oxide ENMs to BEAS-2B cells and murine myeloid (RAW 264.7) cells
[42]	Perturbation model was presented predicting the cytotoxicity of ENMs against several mammalian cell lines; influence of molar volume, polarizability, and size of the particles was indicated
[44]	Models were constructed to predict the cytotoxicity in HaCaT cells of 18 different metal oxide ENMs. The factors of molecular weight, cationic charge, mass percentage of metal elements, individual and aggregation sizes were discussed
[45]	Cytotoxicity of TiO_2_ and ZnO ENMs was modeled by MLR and C4.5 algorithm
[47]	Predictive models were built based on cytotoxicity of different ENMs (with diverse metal cores) in four cell lines (endothelial and smooth muscle cells, monocytes, and hepatocytes)
[48]	Based on random forest regression, developed predictive classification models for cytotoxicity of 18 metal oxide ENMs to HaCaT cells
[49]	Structure-activity relationship models (random forest) were introduced for the toxicity of 24 metal oxide ENMs towards BEAS-2B and RAW 264.7 cell lines
[52]	A classification model was built for 24 metal oxide ENMs based on the dissolution of metals and energy of conduction band (*E*_c_)
Toxicity to *Escherichia coli* (*E. coli*)	
[31]	Global classification models were developed to predict the ecotoxicity of metallic ENMs to different species; classification models were also built for *Danio rerio*, *Daphnia magna*, *Pseudokirchneriella subcapitata*, and *Staphylococcus aureus*
[37]	Using the toxicity dataset of 17 metal oxide ENMs to *E. coli*, models were built with the MLR and partial least squares methods
[38]	Perturbation model was introduced for the prediction of ecotoxicity and cytotoxicity of ENMs; molar volume, electronegativity, polarizability, size of the particles, hydrophobicity, and polar surface area were involved in the model
[43]	A quantitative model was developed based on the toxicity data of 16 metal oxide ENMs to *E. coli* using enthalpy of formation of a gaseous cation (Δ*H*_Me+_) and polarization force (Z/r). The toxicity of 35 other metal oxide ENMs was predicted and depicted in the periodic table
[44]	Models were constructed to predict the toxicity of 17 metal oxide ENMs to *E. coli*. The factors of molecular weight, cationic charge, mass percentage of metal elements, individual and aggregation sizes were discussed
[46]	Toxicity and photo-induced toxicity of 17 metal oxide ENMs to *E. coli* was assessed using a self-written least-squares fitting program
[17]	Predicted the cytotoxicity of 17 metal oxide ENMs to *E. coli* with only one descriptor: enthalpy of formation of a gaseous cation having the same oxidation state as that in the metal oxide structure
[47]	Predictive models were built based on the toxicity of 17 different metal oxide ENMs to *E. coli*
[48]	Based on random forest regression, developed predictive classification models for the toxicity of 17 metal oxide ENMs to *E. coli*
[50]	Estimated the toxicity of 17 metal oxide ENMs to *E. coli* by employing the SMILES-based (simplified molecular input line entry system) optimal descriptors

**Table 2:** Summary of the experimental data of ENMs used in nano-(Q)SAR studies.

Nano-(Q)SAR	Dataset Used	Number of ENMs	Core of ENMs	Tested Organism
[37]	[17]	17	Metal oxide	*Escherichia coli* (*E. coli*)
[43]				
[44]				
[17]				
[47]				
[48]				
[50]				
[30]	[53]	146	Metal oxide	PaCa2 pancreatic cancer cells (PaCa2)
[32]				
[33]				
[35]				
[36]				
[47]				
[51]				
[32]	[54]	50	Metal oxide and quantum dots	Endothelial cells, vascular smooth muscle cells, human HepG2 cells, RAW 264.7 cells
[33]				
[40]				
[47]				
[34]	[34]	18	Metal oxide	HaCaT cells
[44]				
[48]				
[41]	[52]	24	Metal oxide	BEAS-2B cells; RAW 264.7 cells
[49]				
[52]				
[39]	[39]	9	Metal oxide	BEAS-2B cells
[45]	[55]	24 TiO_2_, 18 ZnO ENMs	TiO_2_, ZnO ENMs	Rat L2 lung epithelial cells; rat lung alveolar macrophages
[46]	[46]	17	Metal oxide	*E. coli*
[29]	Others			
[31]				
[38]				
[42]				

**Table 3:** Overview of reported information of the data published by Weissleder et al. [53].

Reference	Method of ENM Characterization	Data Accessibility	ENM Number	Other Information
[53]			146	Molecular weight and structures
[30]	679 one-dimensional (1D), two-dimensional (2D) chemical descriptors of modifiers were calculated using PaDEL-Descriptor (v2.8)	Values of PaCa2 pancreatic cancer cells (PaCa2) cellular uptake were available (unit: number of ENMs per cell)	109	SMILES (simplified molecular input line entry system)
[32]	691 molecular descriptors of modifiers from DRAGON (v5.5), ADRIANA (v2.2) and an in-house modeling software package		108	List of modifiers
[33]	MOE descriptors for modifiers were used, including physical properties, surface areas, atom and bond counts, Kier & Hall connectivity indices, kappa shape indices, adjacency and distance matrix descriptors, pharmacophore feature descriptors, and molecular charges	Values of PaCa2 cellular uptake were available (log_10_[ENM]/cell pM)	109	SMILES
[35]	Hyperchem program (v7) for constructing molecular structure of modifiers; geometry was optimized with the Austin Model 1 (AM1) semiempirical method; DRAGON for descriptor calculation	Values of PaCa2 cellular uptake were available (log_10_[ENM]/cell pM )	109	List of modifiers and SMILES
[36]	A pool of 307 descriptors of modifiers was calculated using Cerius 2 (v4.10), DRAGON 6 and PaDEL-Descriptor (v2.11)	Values of PaCa2 cellular uptake were available (log_10_[ENM]/cell pM)	109	List of modifiers
[47]	174 molecular descriptors for the modifiers (topological, electronic, geometrical, and constitutional) were calculated using Chemistry Development Kit (CDK v1.0.3)		109	List of modifiers, chemical structures and SMILES
[51]	SMILES-based optimal descriptors were used		109	SMILES, correlation weights (CWs) of SMILES attributes (SA)

**Table 4:** Overview of quantum-mechanical and image descriptors of 18 metal oxide ENMs, as retrieved from the study by Gajewicz et al. [34].

Quantum-Mechanical Descriptors	Image Descriptors
Standard enthalpy of formation of metal oxide nanocluster (ΔHfc) Total energy (TE) Electronic energy (EE) Core-core repulsion energy (Core) Solvent accessible surface (SAS) Energy of the highest occupier molecular orbital (HOMO) Energy of the lowest unoccupied molecular orbital (LUMO) Chemical hardness (η) Total softness (S) HOMO-LUMO energy gap (*E*_g_) Electronic chemical potential (μ) Valance band (*E*_v_) Conduction band (*E*_c_) Mulliken’s electronegativity (χ^c^) Parr and Pople’s absolute hardness (Hard) Schuurmann MO shift alpha (Shift) Polarizability derived from the heat of formation (Ahof) Polarizability derived from the dipole moment (Ad)	Volume (V) Surface diameter (*d*_S_) Equivalent volume diameter (*d*_V_) Equivalent volume/surface (*d*_Sauter_) Area (A) Porosity (P_x_) Porosity (P_y_) Sphericity (Ψ) Circularity (f_circ_) Anisotropy ratio (AR_X_) Anisotropy ratio (AR_Y_)

**Table 5:** Toxic data to *Escherichia coli* (*E. coli*) reported by Puzyn et al. [17] and Pathakoti et al. [46] along with corresponding ENM characterization.

Endpoint or Descriptor ^a^	Al_2_O_3_	Bi_2_O_3_	CoO	Cr_2_O_3_	CuO	Fe_2_O_3_	In_2_O_3_	La_2_O_3_	NiO	Sb_2_O_3_	SiO_2_	SnO_2_	TiO_2_	V_2_O_3_	Y_2_O_3_	ZnO	ZrO_2_
Effect concentrations and descriptor information from the study by Puzyn et al. [17]																	
log 1/EC_50_ (mol/L)	2.49	2.82	3.51	2.51	3.2	2.29	2.81	2.87	3.45	2.64	2.2	2.01	1.74	3.14	2.87	3.45	2.15
HoF (kcal/mol)	−8244	−1966	−8800	−2829	−955	−1051	−3088	N/A ^b^	64	−2141	−4118	−2611	−9826	−3193	−11,486	−5307	−9835
TE (au ^c^)	−31,466	−36,108	−17,007	−20,104	−45,632	−6971	−40,745	N/A	−28,053	−18,039	−21,060	−41,962	−31,518	−26,083	−30,634	−23,158	−23,405
EE (au)	−63,0309	−695,663	−298,812	−307,815	−874,569	−44,000	−872,315	N/A	−432,596	−221,602	−321,879	−874,369	−576,824	−441,766	−511,019	−379,005	−358,169
Core (au)	598,843	659,555	281,806	287,711	828,937	37,029	831,570	N/A	404,543	203,563	300,818	832,407	545,306	415,683	480,385	355,847	334,764
CA (A^2)	1109	1551	1072	659	639	243	1314	N/A	659	975	753	1734	1100	1130	1805	855	1055
CV (A^3)	2260	4107	1548	1161	1108	319	3095	N/A	1088	1797	1467	3959	2340	2426	5401	1849	2403
HOMO (eV)	−4.9	−4.1	−10.5	−6.9	−6.1	−7.1	−8.2	N/A	−5.8	−8.3	−7.1	−6.1	−10.3	−3.5	−1.3	−10.8	−6.2
LUMO (eV)	−0.29	−1.4	−8.28	−0.49	−2.25	−0.68	−3.37	N/A	−1.03	−1.03	−3.89	−2.29	−2.86	0.64	1.2	−6.89	−4.54
GAP (eV)	−4.59	−2.71	−2.2	−6.41	−3.85	−6.45	−4.79	N/A	−4.73	−7.27	−3.23	−3.85	−7.47	−4.17	−2.48	3.87	−1.65
Δ*H*_Clust_ (kcal/mol)	−8017	−1601	−8318	−2264	−759	−140	−3190	N/A	325	−1526	−3295	−2091	−8731	−3157	−11,485	−5357	−8956
Δ*H*_Me+_ (kcal/mol)	1188	1137	602	1269	706	1408	1271	1017	597	1233	1686	1717	1576	1098	837	662	1358
Δ*H*_L_ (kcal/mol)	−3695	−3199	−933	−3645	−992	−3589	−3449	−2969	−965	−3281	−3158	−2821	−2896	−3555	−3111	−971	−2641
Descriptor information from the study by Kar et al. [37]																	
χ	1.61	2.02	1.88	1.66	1.9	1.83	1.78	1.1	1.91	2.05	1.9	1.96	1.54	1.63	1.22	1.65	1.33
∑χ	3.22	4.04	1.88	3.32	1.9	3.66	3.56	2.2	1.91	4.1	1.9	1.96	1.54	3.26	2.44	1.65	1.33
∑χ/nO	1.073	1.347	1.880	1.107	1.900	1.220	1.187	0.733	1.910	1.367	0.95	0.98	0.77	1.087	0.813	1.650	0.665
MW	102.0	466.0	74.9	152.0	79.5	159.6	277.6	325.8	74.7	291.5	60.1	150.7	79.9	149.9	225.8	81.4	123.2
*N*_Metal_	2	2	1	2	1	2	2	2	1	2	1	1	1	2	2	1	1
*N*_Oxygen_	3	3	1	3	1	3	3	3	1	3	2	2	2	3	3	1	2
χ*_ox_*	3	3	2	3	2	3	3	3	2	3	4	4	4	3	3	2	4
Descriptor information from the studies of Singh and Gupta [47] and Toropov et al. [50]																	
SMILES notation	O = [Al]O[Al] = O	O = [Bi]O[Bi] = O	[Co] = O	O = [Cr]O[Cr] = O	[Cu] = O	O = [Fe]O[Fe] = O	O = [In]O[In] = O	O = [La]O[La] = O	[Ni] = O	O = [Sb]O[Sb] = O	O = [Si] = O	O = [Sn] = O	O = [Ti] = O	O = [V]O[V] = O	O = [Y]O[Y] = O	O = [Zn]	O = [Zr] = O
Descriptor information from the study by Sizochenko et al. [48]																	
Size (nm)	44	90	100	60	N/A	32	30	46	30	20	150	15	46	15	38	71	47
Aggregation size (nm)	372	2029	257	617	N/A	298	224	673	291	223	640	810	265	1307	1223	189	661
Effect concentrations and descriptor information from the study by Pathakoti et al. [46]																	
toxicity under darkness, log 1/EC_50_ (mol/L)	2.42	3.55	3.13	2.06	4.24	2.4	2.83	4.96	3.79	3.12	2.54	2.53	2.14	3.48	5.79	5.8	2.58
toxicity under sunlight exposure, log 1/EC_50_ (mol/L)	2.75	4.02	3.33	2.06	5.71	2.54	3.48	5.56	3.87	3.66	2.92	3.24	4.68	3.78	5.84	6.23	3.04
Particle size (vendor) (nm)	<50	90–210	<100	<100	<50	<50	<100	<100	<50	90–210	10–20	<100	<100	N/A	<50	<100	<100
Particle size TEM (nm)	55 ± 17	144 ± 7	55 ± 13	47 ± 27	28 ± 7	68 ± 20	60 ± 14	65 ± 19	14 ± 9	84 ± 23	20 ± 5	15 ± 4	42 ± 9	N/A	38 ± 9	71 ± 17	27 ± 6
Hydrodynamic size (nm)	330	4084	262	426	285	>6000	308	508	399	619	1230	3971	748	307	357	1614	2337
Zeta potential (mV) (H_2_O)	30.3 ± 1.3	−(16.5 ± 0.8)	17.5 ± 1.5	−(12.0 ± 1.3)	24.4 ± 0.6	−(6.3 ± 1.0)	22.6 ± 0.4	−(3.6 ± 1.1)	26.0 ± 0.4	−20.7 ± 1.3	−29.8 ± 1.9	−21.1 ± 0.4	−(10.7 ± 2.5)	−(27.9 ± 0.9)	16.3 ± 0.9	−(20.9 ± 0.5)	−(6.9 ± 0.5)
Zeta potential (mV) (KCl)	25.3 ± 1.1	−(4.9 ± 0.1)	26.0 ± 0.5	23.3 ± 1.0	19.1 ± 0.3	−(19.5 ± 1.9)	28.7 ± 0.4	22.3 ± 1.7	26.8 ± 1.2	−(12.7 ± 0.4)	−(33.7 ± 1.6)	−(16.7 ± 0.2)	−(2.2 ± 0.4)	−(32.6 ± 0.5)	17.9 ± 1.0	−(24.9 ± 0.3)	4.0 ± 2.7
Surface area (m^2^/g)	37	N/A	>8	N/A	33	36	28	20	80	N/A	N/A	18.6	36	N/A	31	15	22
HHOMO (au)	−0.283	−0.253	−0.221	−0.245	−0.236	−0.283	−0.265	−0.187	−0.241	−0.262	−0.343	−0.305	−0.265	−0.219	−0.189	−0.228	−0.243
LZELEHHO (au)	0.211	0.184	0.169	0.199	0.178	0.175	0.196	0.121	0.180	0.174	0.245	0.224	0.195	0.174	0.129	0.132	0.184
LUMOA (au)	−0.138	−0.116	−0.116	−0.152	−0.121	−0.066	−0.127	−0.054	−0.120	−0.086	−0.147	−0.143	−0.125	−0.129	−0.068	−0.036	−0.125
LUMOB (au)	−0.138	−0.116	−0.131	−0.117	−0.119	−0.163	−0.127	−0.054	−0.114	−0.086	−0.147	−0.143	−0.125	−0.106	−0.068	−0.139	−0.125
ALZLUMO (au)	−0.138	−0.116	−0.123	−0.135	−0.120	−0.114	−0.127	−0.054	−0.117	−0.086	−0.147	−0.143	−0.125	−0.117	−0.068	−0.087	−0.125
*Cp* (J mol^−1^ K^−1^)	79.04	113.51	55.23	118.74	42.3	103.85	92	108.78	44.31	101.63	44.43	52.59	55.48	103.22	102.51	40.25	56.19
MHOMOA (au)	−0.218	−0.319	−0.232	−0.222	−0.289	−0.229	−0.202	−0.188	−0.236	−0.334	−0.301	−0.267	−0.232	−0.247	−0.211	−0.293	−0.232
MLUMOA (au)	0.017	0.114	0.036	0.027	0.036	0.031	0.010	0.015	0.035	0.130	−0.007	−0.017	0.021	0.024	0.018	0.043	0.016
QMELECT (au)	0.101	0.103	0.098	0.097	0.126	0.099	0.096	0.086	0.101	0.102	0.154	0.142	0.106	0.111	0.097	0.125	0.108

**^a^** EC50—the effective concentration that causes 50% response; HoF—the standard heat of formation of the oxide cluster; TE—total energy of the oxide cluster; EE—electronic energy of the oxide cluster; Core—core-core repulsion energy of the oxide cluster; CA—area of the oxide cluster calculated based on COSMO; CV—volume of the oxide cluster calculated based on COSMO; HOMO—energy of the highest occupier molecular orbital of the oxide cluster; LUMO—energy of the lowest unoccupied molecular orbital of the oxide cluster; GAP—energy difference between HOMO and LUMO energies; Δ*H*_Clust_—enthalpy of detachment of metal cations Me^n+^ from the cluster surface; Δ*H*_Me+_- enthalpy of formation of a gaseous cation; Δ*H*_L_—lattice energy of the oxide; ^b^ N/A—data not available; ^c^ au—atomic units.

**Table 6:** Overview of computational descriptors or factors discussed in nano-(Q)SAR studies, including information on the original dataset for modeling. Name of the descriptors in original publications are given in the parenthesis (if available).

Reference	Descriptor or Identified Factor by Developed Models	Dataset
Studies of modeling cellular take of ENMs		
[30]	Number of CH_2_ groups, primary, secondary and tertiary nitrogen, halogens (fluorine, bromine, iodine), sulfur atoms, fused rings, hydrogen bonding	[53]
[32]	Number of 10 membered rings (nR10), molecular asphericity (ASP), d COMMA2 value/weighted by atomic masses (DISPm), Qzz COMMA2 value/weighted by atomic masses (QZZm), number of secondary amides, aliphatic (nRCONHR), number of (thio-) carbamates, aromatic (nArOCON), CH3X (C-005), number of circuits (nCIR), number of N atoms (nN), average molecular span R (SPAM), Qyy COMMA2 value/weighted by atomic polarizabilities (QYYp), number of total secondary C sp^3^ (nCs), number of aromatic hydroxyls (nArOH), H attached to C0(sp^3^) with 2X attached to next C (H-053), =O (O-058)	
[33]	Surface area “owned” with SlogP weight −10 to −0.40 (SlogP_VSA0), surface area “owned” with SlogP weight −0.40 to −0.20 (SlogP_VSA1), surface area “owned” with SlogP weight −0.20 to 0 (SlogP_VSA2), surface area “owned” with SlogP weight −0.15 to −0.20 (SlogP_VSA5), van der Waals surface area surface area of hydrogen-bond donors (vsa_don), van der Waals surface area of nondonor/-acceptor atoms (vsa_other), van der Waals surface area surface area of basic atoms (vsa_base), sum of the van der Waals surface area of atoms whose PEOE partial charge is positive, divided by the total surface area (PEOE_VSA_FPOS), van der Waals surface area where atomic partial charge 0.05 < q < 0.10 (PEOE_VSA+1), number of double bonds, aromatic bonds are not considered (b_double)	
[35]	Number of donor atoms for H-bonds (nHDon), Geary autocorrelation of lag 1 weighted by van der Waals volume (GATS1v), 3D-MoRSE-signal 29/unweighted (Mor29u), D total accessibility index/weighted by Sanderson electronegativity (De), 3D-MoRSE-signal 14/unweighted (Mor14u), mean electrotopological state (Ms)	
[36]	Hydrophobicity of the N atom in primary aliphatic amine (Al-NH_2_) fragment (*Atype*-N-66), hydrophobicity of the N atom in a secondary aliphatic amine (Al_2_-NH) fragment (*Atype*-N-67), measure of electronic features of the molecule relative to molecular size (∑β'), relative positive charge surface area (*Jurs*-*RPCS*), all-path Wiener index (*Wap*), number of aliphatic nitro groups (*nRNO*2)	
[47]	Weighted partial negative surface area-3 (WNSA-3), weighted partial positive area-2 (WPSA-2), Chi simple path descriptor of order 5 (SP-5), Chi valance path descriptor of order 4 (VP-4), moment of inertia along X/Z-axis (MOMI-XZ), logarithmic form of octanol-water partition coefficient predicted by atomic method (XlogP), number of rotatable bonds (nRotB), number of hydrogen bond donors (nHBDon), Chi valance path cluster of order 6 (VPC-6), ionization potential (IP), number of hydrogen acceptors (nHBAcc)	
Studies of modeling cytotoxicity of ENMs to cell lines		
[34]	Enthalpy of formation of metal oxide nanocluster representing a fragment of the surface (∆*H_f_*^c^), Mulliken’s electronegativity of the nanocluster (χ^c^)	[34]
[44]	Molecular weight, cationic charge, mass percentage of metal elements, individual size, aggregation size	
[48]	Unbonded two-atomic fragments [Me]···[Me] (*S*_1_), Wigner-Seitz radius of oxide’s molecule (*r*_w_), mass density (*ρ*), covalent index of the metal ion (*CI*), SiRMS-derived number of oxygen’s atoms in a molecule (*S*_2_), aggregation parameter (*AP*)	
[32]	Core material (*I*_Fe3O4_), surface coating (*I*_dextran_), surface charge (*I*_surf.chg_)	[54]
[33,40,47]	Size, R1 relaxivity, R2 relaxivity, zeta potential	
[52]	Conduction band energy (*E*_c_), solubility of metals	[52]
[41]	Ionic index of metal cation (*Z*^2^/*r*), ENM conduction band energy (*E*_c_), metal oxide ionization energy (∆*H*_IE_), metal oxide electronegativity (χ_MeO_), atomization energy of metal oxide (*E*_Amz_), primary size (*d*), atomic mass of ENM metal (*m*_Me_)	
[49]	Mass density, molecular weight, aligned electronegativity, covalent index, cation polarizing power, Wigner-Seitz radius, surface area, surface-area-to-volume ratio, aggregation parameter, two-atomic descriptor of van der Waals interactions, tri-atomic descriptor of atomic charges, tetra-atomic descriptor of atomic charges, size in DMEM	
[45]	Size of ENMs (X0), size in water (X1), size in phosphate buffered saline (X2), concentration (X4), zeta potential (X5)	[55]
[39]	Size of ENM (*d*), volume concentration (*θ*_v_), period of the ENM metal in the periodic table (*P*_Me_), atomization energy of the metal oxide (*E*_MeO_)	[39]
[42]	Molar volume, polarizability, size of ENMs, electronegativity, hydrophobicity and polar surface area of surface coatings	Others
Studies of modeling the toxicity of ENMs to species		
[46]	Absolute electronegativity of the metal atom (QMELECT), absolute electronegativity of the metal oxide (LZELEHHO), literature molar heat capacity of the metal oxide at 298.15 K (*Cp*), average of the alpha and beta LUMO energies of the metal oxide (ALZLUMO)	[46]
[17]	Enthalpy of formation of a gaseous cation having the same oxidation state as that in the metal oxide structure (∆*H*_Me+_)	[17]
[37]	Charge of the metal cation corresponding to a given oxide (χ*_ox_*), metal electronegativity (χ)	
[43]	Enthalpy of formation of a gaseous cation having the same oxidation state as that in the metal oxide structure (∆*H*_Me+_), polarization force (Z/*r*)	
[44]	Molecular weight, cationic charge, mass percentage of metal elements, individual size, aggregation size	
[47]	Oxygen percent, molar refractivity, polar surface area	
[48]	Unbonded two-atomic fragments [Me]···[Me] (*S*_1_), Wigner-Seitz radius of oxide’s molecule (*r*_w_), mass density (*ρ*), cation polarizing power (*CPP*), SiRMS-derived number of oxygen’s atoms in a molecule (*S*_2_), tri-atomic fragments [Me]-[O]-[Me] (*S*_3_), proportion of surface molecules to molecules in volume (*SV*)	
[31]	Molecular polarizability, accessible surface area, solubility	Others
[38]	Molar volume, polarizability, size of ENMs, electronegativity, hydrophobicity and polar surface area of surface coatings	Others
